# Genome-scale dissection of phase-variable gene function in *Campylobacter jejuni* using a stabilized phasotype library

**DOI:** 10.1128/msphere.00064-26

**Published:** 2026-06-10

**Authors:** Shouji Yamamoto, Ken-ichi Lee, Akiko Kubomura, Sunao Iyoda, Yukihiro Akeda, Takaaki Shimohata, Chihiro Aikawa, Masashi Okamura, Fuhito Hojo, Takako Osaki, Jiro Mitobe

**Affiliations:** 1 Department of Bacteriology I, National Institute of Infectious Diseases, Japan Institute for Health Security 739298 , Shinjuku City, Tokyo, Japan; 2 Faculty of Marine Biosciences, Fukui Prefectural University 12767, Fukui, Japan; 3 Division of Veterinary Science, Department of Veterinary Medicine, Obihiro University of Agriculture and Veterinary Medicine 52746, Obihiro, Hokkaido, Japan; 4 Graduate School of Medicine, Institute of Laboratory Animals, Kyorin University 12912, Mitaka, Tokyo, Japan; 5 Department of Infectious Diseases, Kyorin University School of Medicine, Mitaka, Tokyo, Japan; Meharry Medical College, Galveston, Texas, USA

**Keywords:** *Campylobacter jejuni*, phase variation, host adaptation, genome editing

## Abstract

Phase variation (PV) allows bacterial pathogens to rapidly modify surface structures through reversible mutations—often driven by simple sequence repeats (SSRs)—thereby facilitating immune evasion and environmental adaptation. In *Campylobacter jejuni*, SSR-mediated PV regulates numerous surface-exposed molecules, including capsular polysaccharides and lipooligosaccharides, which play key roles in host interaction and immune resistance. However, the stochastic nature of PV has made it difficult to systematically analyze the functions of individual phase-variable genes (PVGs). Here, we introduce PV-GenShift (Phase Variation Genomic Shift), a genome-scale screening platform built on a genetically stabilized library of phase-locked *C. jejuni* variants. Using multiplex genome editing via natural transformation, we fixed the ON/OFF states of 15 SSR-containing PVGs, enabling reproducible, high-resolution analysis of phasotypes, defined as unique combinations of ON/OFF states across multiple PVGs, under defined selective pressures. Applying PV-GenShift to models of human serum exposure, murine colonization, and chicken gut passage, we identified distinct phasotypes linked to serum resistance and enrichment during mouse colonization—particularly those involving capsular polysaccharide modifications such as *O*-methyl phosphoramidation and methylation. In contrast, chicken passage produced heterogeneous ON/OFF patterns without a dominant phasotype. These findings underscore the combinatorial influence of SSR-mediated PVG expression states on bacterial adaptation and establish PV-GenShift as a versatile framework for dissecting PV-driven phenotypic diversity. This approach offers a scalable strategy for mapping genotype-phenotype relationships and provides insights with implications for vaccine development, diagnostic design, and targeted therapeutics.

**IMPORTANCE:**

Phase variation allows bacteria to rapidly generate phenotypic diversity, but its randomness has long limited efforts to define how specific gene combinations influence adaptation. In *Campylobacter jejuni*, phase-variable genes control numerous surface structures that affect host interactions and immune resistance, yet their coordinated contributions remain poorly understood. PV-GenShift (Phase Variation Genomic Shift) overcomes this challenge by genetically stabilizing the ON/OFF states of multiple phase-variable genes, enabling systematic, reproducible analysis of phasotypes under defined selective pressures. Using this approach, we identified combinations of gene states that enhance serum resistance and promote colonization in mice, particularly those involving modifications to capsular polysaccharides. In contrast, chicken passage produced heterogeneous patterns consistent with weak or non-specific selection. These findings demonstrate that the interplay of multiple phase-variable loci shapes host adaptation and highlight PV-GenShift as a broadly applicable strategy for dissecting phase variation in diverse pathogens, with implications for vaccine design and targeted therapies.

## INTRODUCTION

Bacterial pathogens employ multiple strategies to survive in dynamic environments, one of the most prominent being phase variation (PV)—a reversible, high-frequency mechanism of gene regulation that enables clonal populations to generate phenotypic diversity without altering their core genome (1, 2). Such stochastic switching in gene expression promotes immune evasion (3–6), pathogenicity (7, 8), environmental adaptation (9), and resistance to antibiotics and bacteriophages (10–12). PV frequently targets surface structures, including pili, flagella, outer membrane proteins, and polysaccharides, all of which are essential mediators of host–pathogen interactions (1, 13–15).

**Fig 1:**
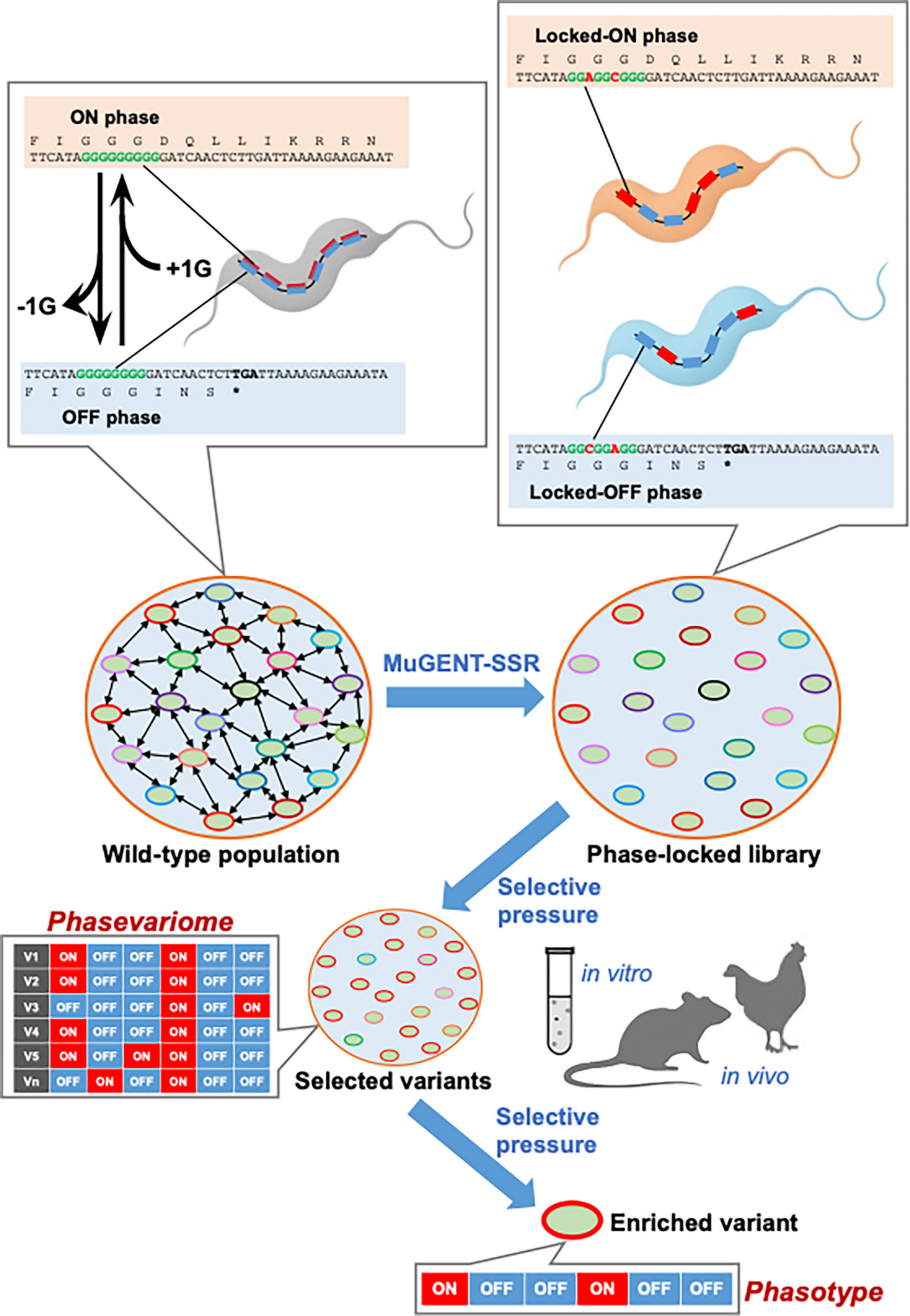
Schematic overview of the PV-GenShift strategy and phasevariome-guided screening. PV-GenShift is a stepwise screening framework that utilizes a genetically stabilized phase-locked library of *C. jejuni* variants. PVGs containing polyG/C tracts were locked in either the ON/OFF phase using MuGENT-SSR, eliminating stochastic switching and enabling the controlled analysis of phasotypes. In this schematic, six PVGs are shown as an illustrative example, with ON-phase genes indicated in red and OFF-phase genes in blue. This configuration allows for the theoretical generation of 2⁶ (= 64) distinct phasotypes, but does not represent the full diversity of the phase-locked library. Unlike wild-type populations, which are genetically and phenotypically unstable due to spontaneous phase variation, the phase-locked library provides a highly stable platform for investigating the functional roles of specific phasotypes under defined selective pressures. The phase-locked library was subjected to biologically relevant selective pressures both *in vitro* and *in vivo* (in this study, human serum exposure, colonization in IL-10-knockout mice, and chicken gut passage). Phasevariome analysis of recovered populations revealed distinct ON/OFF expression patterns across PVGs, enabling identification of enriched phasotypes. Once a dominant phasotype is identified, functional validation is performed by switching individual ON-phase genes to the OFF phase to assess their contribution to the observed phenotype. This allows precise identification of causal PVGs involved in host adaptation.

A major driver of PV is simple sequence repeats (SSRs), which are prone to slipped-strand mispairing during replication. This process generates insertions or deletions that disrupt coding or regulatory regions, producing ON/OFF switching or modulation of gene expression (16, 17) (Fig. 1). SSR-mediated PV is well documented across diverse species, including *Neisseria* spp., *Haemophilus influenzae*, *Helicobacter pylori*, and *Campylobacter jejuni* (18–22). Additional PV mechanisms include promoter inversion, as seen in *Salmonella* (23, 24), and epigenetic regulation that coordinates “phasevarions” of co-regulated genes (25).

*C. jejuni*, a major cause of gastroenteritis and Guillain-Barré syndrome (GBS), exhibits striking clinical variability ranging from mild diarrhea to severe bloody diarrhea (26). This heterogeneity is thought to arise in part from extensive genomic variability and PV. SSR-mediated PV regulates genes involved in the biosynthesis and modification of lipooligosaccharides (LOS), capsular polysaccharides (CPS), and flagella (27–35), thereby influencing colonization and immune evasion (36–44). Notably, LOS PV can generate host ganglioside mimics, contributing to GBS pathogenesis (32), and complicating vaccine development (33, 45, 46).

Combinatorial ON/OFF states of phase-variable genes (PVGs)—referred to as phasotypes (47)—affect phenotypes such as serum resistance and phage susceptibility (10, 28, 48). Host passage reshapes the phasevariome, the full set of PVGs and their current expression states within a population, influencing adaptation and population structure (47, 49–51). However, the stochastic nature of PV, coupled with the presence of 18–39 hypermutable SSRs per strain, makes experiments difficult to reproduce and obscures causal links between PVGs and phenotypic outcomes (52).

To overcome these challenges, we developed PV-GenShift (Phase Variation Genomic Shift), a genome-scale strategy that locks polyG/C-associated PVGs into defined ON/OFF states, generating a genetically stabilized variant library for controlled experimental evolution under selective pressures (Fig. 1). Applied to *C. jejuni*, PV-GenShift enables systematic dissection of PV-driven adaptation and provides a broadly applicable platform for investigating the functional consequences of phase variation across diverse pathogens.

## RESULTS

### Experimental design of PV-GenShift

PV-GenShift is a screening strategy built on a genetically stabilized library of *C. jejuni* phase-locked variants. By fixing the ON/OFF states of PVGs, the platform enables systematic evaluation of PVG contributions under defined selective pressures. The stepwise workflow allows isolation of phenotypes that are typically transient or obscured by population heterogeneity (Fig. 1).

#### Library construction and validation

Multiplex genome editing was used to lock polyG/C SSRs within coding regions into either ON/OFF states, generating a diverse and genetically stable set of phasotypes. Whole-genome sequencing (WGS) verified the precision of editing and confirmed that the resulting ON/OFF configurations produced consistent, stable expression profiles.

#### Stepwise screening under selective pressures

The stabilized library was subjected to three selective environments that mimic host-associated pressures:

- Human serum, assessing complement resistance
- Mouse colonization, probing intestinal fitness
- Chicken gut passage, examining host-specific adaptation

This stepwise screening enriches variants with advantageous phasotypes and can be readily adapted for other selective contexts, including exposure to antimicrobials or bacteriophages.

#### Phasevariome analysis

WGS of recovered populations enabled characterization of the phasevariome, defined as the complete set of PVG expression states present in the population. Because the PV-GenShift platform relies on a genetically phase-locked library, changes in ON/OFF profiles observed across screening conditions reflect selection acting on pre-existing phasotypes rather than *de novo* PV events. Consequently, phasevariome comparisons across conditions are reproducible and allow direct attribution of adaptive shifts to specific PVG configurations.

#### Phasotype dissection

Dominant phasotypes enriched under selection were identified, and the ON-state PVGs within these phasotypes were examined to determine their contributions to specific adaptive traits.

### Construction and evaluation of a phase-locked variant library in *C. jejuni* strain 81-176

To enable systematic analysis of PVGs, we constructed a genetically stabilized library based on *C. jejuni* strain 81-176. This strain contains 18 polyG/C SSR tracts, of which 15 are located within open reading frames and are associated with PVG expression (52) (Table 1). Although homopolymeric A/T tracts and more complex SSRs (e.g., di- or trinucleotide repeats) are present in *C. jejuni*, these elements are comparatively infrequent and are not typically associated with classical contingency loci or high-frequency phase variation and were therefore excluded from the present analysis (21, 53, 54). The remaining three polyG/C tracts are located in intergenic regions and were excluded because SSRs in promoter or Shine-Dalgarno contexts are expected to exert context-dependent, quantitative effects on gene expression rather than discrete frameshift-mediated ON/OFF outcomes (17). Thus, a defined set of 15 polyG/C SSR-associated PVGs was selected as the target set for systematic analysis. Among the 15 PVGs, *CJJ81176_1421* was treated as a single PVG despite overlapping annotation with *CJJ81176_1420*, based on a clear Shine-Dalgarno sequence upstream of *CJJ81176_1421* and shared reading frame (Fig. S1), suggesting *CJJ81176_1420* is misannotated.

**TABLE 1:** Predicted products of polyG/C-containing PVGs stabilized by MuGENT-SSR in *C. jejuni* 81-176^*c*^

PVG	SSR (repeats in ON phase^*a*^)	Product^*b*^		
		Predicted function	Predicted amino acid residues	
			ON phase	OFF phase
*CJJ81176_0086*	G (9)	Anion transporter	59	30
*CJJ81176_0206*	G (9)	Hypothetical	229	66
*CJJ81176_0646*	G (9)	Hypothetical	409	203
*CJJ81176_0708*	C (9)	Invasion protein	464	285
*CJJ81176_0758*	G (9)	Hypothetical	213	207
*CJJ81176_1160*	G (10)	Beta-1,4-*N*-acetylgalactosamine transferase involved in LOS biosynthesis (CgtA)	315	205
*CJJ81176_1312*	G (9)	Dimethylglyceric acid modification	252	57
*CJJ81176_1325*	G (9)	Formyl transferase domain protein	136	72
*CJJ81176_1327*	G (9)	Hypothetical	403	204
*CJJ81176_1341*	G (9)	Hypothetical	416	196
*CJJ81176_1419*	G (9)	Putative CPS methyltransferase	257	145
*CJJ81176_1421*	G (9)	*O*-Methyl phosphoramidate transferase to C4-galactose of CPS	592	48
*CJJ81176_1429*	G (10)	Hypothetical	307	101
*CJJ81176_1432*	G (9)	Galactosyltransferase	569	564
*CJJ81176_1435*	G (9)	*O*-Methyl phosphoramidate transferase to C2-galactose and C6-galactose of CPS	603	34

^*a*^ The ON phase refers to the SSR repeat length that yields the longest in-frame translation product, whereas the OFF phase corresponds to a −1 nucleotide deletion that causes a frameshift and premature termination.

^*b*^ Predicted functions of the PVGs were annotated based on Wanford et al. (49).

^*c*^ MuGENT-SSR, Multiplex Genome Editing by Natural Transformation against SSRs.

SSR tracts within coding regions undergo slipped-strand mispairing during replication, producing reversible frameshift mutations that switch genes between ON and OFF states. We defined the ON phase as the SSR length yielding the longest in-frame product, and the OFF phase as the −1 nucleotide frameshift variant (Table 1). Across 15 PVGs, this architecture theoretically permits up to 2¹⁵ distinct phasotypes per genome.

**Fig 2:**
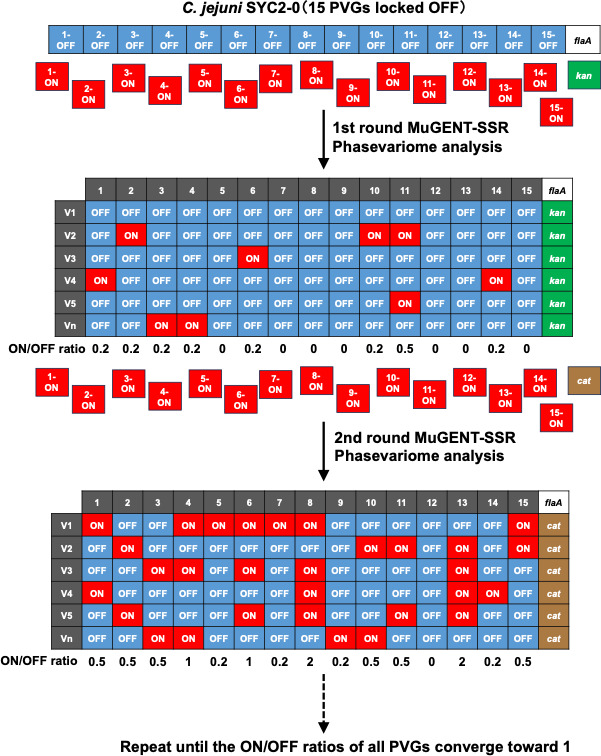
Construction and evaluation of a phase-locked variant library in *C. jejuni* strain 81-176. In the first round of MuGENT-SSR, the *C. jejuni* 81-176 variant SYC2-0, in which all 15 PVGs were locked in the OFF phase, was used as the starting strain. A total of 15 PVGs containing polyG/C tracts within coding regions were genetically stabilized using MuGENT-SSR. Each PVG was locked in either the ON/OFF phase, resulting in a diverse library of phase-locked variants. The ON phase corresponds to an SSR length that maintains the correct reading frame for full-length protein translation, whereas the OFF phase is induced by a −1 nucleotide frameshift, leading to premature termination. Phasotypes of variants (V1–Vn) are shown as illustrative examples, with ON-phase genes indicated in red and OFF-phase genes in blue. These examples highlight the combinatorial diversity of PVG expression states across the library. MuGENT-SSR was performed in sequential rounds using selected DNA fragments carrying antibiotic resistance markers (kanamycin or chloramphenicol) targeted to *flaA*, along with unselected DNA fragments designed to lock PVGs in the ON state. Whole-genome sequencing of pooled colonies (20,000–30,000 per round) was used to assess editing efficiency and phase distribution. ON/OFF ratios for each PVG were calculated using PVfinder_81176, a BLAST-based computational tool developed for this study. The ON/OFF ratio shown below the table was calculated from six data points (V1 through Vn) as an illustrative example. The editing process was repeated until the ON/OFF ratios of all PVGs converged toward 1, indicating a balanced and complete phasevariome suitable for downstream screening.

We generated a genetically stable population using MuGENT-SSR (Multiplex Genome Editing by Natural Transformation against SSRs), a high-efficiency method based on natural transformation and co-transformation (55) (Fig. 2). Two donor DNA types were used: (i) selected DNA with an antibiotic marker (kanamycin/chloramphenicol) inserted into a neutral locus (i.e., *flaA*), and (ii) unselected DNA comprising 15 fragments introducing mutations into polyG/C tracts, locking each gene ON/OFF. Different antibiotic markers were used in each MuGENT cycle to maintain efficiency. Sequential rounds progressively increased incorporation of locked mutations, ensuring all PVGs were edited (Fig. 2).

Initially, all PVGs were locked OFF by co-transforming cells with selected DNA and unselected DNA carrying locked-OFF mutations (e.g., GGG GGG GGG → GGA GGC GGG) (Fig. S2). Antibiotic selection yielded a population with all PVGs stably OFF. A second MuGENT-SSR round introduced locked-ON mutations (e.g., GGG GGG GGG → GGC GGA GGG), randomly converting subsets to ON. Repeating this step generated a collection of phase-locked variants with distinct phasotypes (Fig. 2). All serial rounds of MuGENT-SSR were performed sequentially using a single parental culture. Each round of transformation was carried out on the population obtained from the preceding round, rather than on independently derived cultures, to generate a controlled baseline phase-locked library.

To assess editing completeness, short-read WGS was performed on pooled genomic DNA (gDNA) from ~20,000–30,000 colonies post-MuGENT-SSR. Sequencing data were analyzed using PVfinder_81176, a BLAST-based tool developed for this study. PVfinder_81176 calculates ON/OFF ratios for each PVG by comparing reads for locked-ON and locked-OFF alleles (Table S6). Ratios near 1 indicate balanced representation; ∞ or 0 indicate ON/OFF dominance, respectively. The ON/OFF ratio profile—phasevariome—was used to select libraries with complete, well-distributed phasotypes (Fig. 2). Five iterative MuGENT-SSR rounds improved mutation coverage (Table 2; Table S7). Phasevariome analysis showed variable ON/OFF shifts: some genes reached near-equilibrium by round 3, others remained OFF-biased (<0.3) even after round 5. The fifth-round population was designated PLL_81176. To restore motility disrupted by *flaA* cassette insertion, *flaA* was repaired via natural transformation with unmarked donor DNA, reconstituting functional flagella. The final motile library, PLL_81176M, comprised ~20,000 colonies, encompassing diverse phasotypes for phenotypic screening. Although selection shifted the phasevariome for several loci, the ON-state proportion of the majority of PVGs remained below 0.5. This reflects the non-uniform composition of the starting phase-locked library, in which certain loci remain OFF-biased due to locus-specific differences in editing efficiency and the cumulative effects of sequential MuGENT-SSR cycles.

**TABLE 2:** Phasevariome profiles during the construction of the phase-locked library in *C. jejuni* strain 81-176

PVG	ON/OFF ratio^*a*^ of PVGs across MuGENT-SSR round					
	0	1	2	3	4	5
*CJJ81176_0086*	0	0.10	0.12	0.17	0.23	0.27
*CJJ81176_0206*	0	0.20	0.30	0.34	0.40	0.44
*CJJ81176_0646*	0	0.08	0.12	0.16	0.13	0.27
*CJJ81176_0708*	0	0.17	0.18	0.25	0.33	0.38
*CJJ81176_0758*	0	0.09	0.15	0.17	0.21	0.32
*CJJ81176_1160*	0	0.24	0.31	0.91	1.01	1.50
*CJJ81176_1312*	0	0.11	0.10	0.12	0.19	0.35
*CJJ81176_1325*	0	0.20	0.21	0.29	0.55	0.41
*CJJ81176_1327*	0	0.14	0.19	0.34	0.40	0.52
*CJJ81176_1341*	0	0.06	0.12	0.15	0.26	0.23
*CJJ81176_1419*	0	0.15	0.14	0.69	0.62	1.08
*CJJ81176_1421*	0	0.05	0.02	0.07	0.15	0.23
*CJJ81176_1429*	0	0.33	0.48	0.74	1.97	0.93
*CJJ81176_1432*	0	0.27	0.32	0.51	0.53	0.31
*CJJ81176_1435*	0	0.08	0.10	0.56	0.36	0.91

^*a*^ PVfinder_81176 calculated the ON/OFF ratio for each PVG by comparing the number of reads corresponding to locked ON and locked OFF alleles.

To characterize the structure of the phase-locked library, single-colony phasotyping was performed on 24 colonies randomly sampled from the library (Fig. S3). This analysis revealed that the library is not dominated by variants carrying single PVGs in the ON state but instead is enriched for intermediate- to high-order phasotypes comprising multiple ON loci (Fig. S4).

### Systematic phasotype screening identifies determinants of human serum resistance in PLL_81176M

*C. jejuni* can exhibit resistance to multiple antimicrobial pressures, including components of human serum, selected antimicrobial peptides, and bacteriophages; however, the extent of this resistance is highly dependent on the bacterial strain and the specific challenge encountered. In the case of serum, resistance has primarily been demonstrated using normal human serum. Similarly, susceptibility to antimicrobial peptides and bacteriophages varies according to the molecular properties of the host factor and the composition of strain-specific surface structures. Surface antigens such as CPS and LOS are critical for these defenses (10, 28, 38, 39, 56), facilitate host cell invasion (29, 38, 57), and are linked to diseases including diarrhea and GBS (39, 41, 42, 58). Since PV drives antigen diversity, understanding its role in immune evasion is essential.

To evaluate PV-GenShift, we performed screening and phasevariome analysis using serum resistance as a model. For phasevariome analysis of the serum resistance screens, gDNA for WGS was extracted from pooled bacterial populations corresponding to approximately 10,000 colonies from the pre-exposure input inoculum and approximately 3,000 colonies recovered from the surviving output population after the first round of serum exposure. In subsequent selection rounds, gDNA was extracted from pooled output populations corresponding to approximately 5,000 colonies per round. Cultures of PLL_81176M (input) were exposed to human serum; survivors (output) were re-exposed in subsequent rounds. No colonies survived in the *kpsE* mutant lacking CPS (57) (Table S1, SYC2005). Serum resistance increased 33-fold by the third round, compared to 13.5 ± 4.6% for the parental strain.

Phasevariome analysis revealed >10-fold ON increases in *CJJ81176_0758, CJJ81176_1419*, *CJJ81176_1421*, and *CJJ81176_1435*, while *CJJ81176_1429* shifted OFF (Table 3; Table S8). Five colonies from the third output yielded three distinct phasotypes: PT1211, PT4263, and PT9397, with PT9397 being the most resistant (Table 4). Switching ON-phase genes in PT9397 to OFF showed serum resistance required *CJJ81176_1419*, *CJJ81176_1429*, and *CJJ81176_1435* (PT21) (Table 4). *CJJ81176_1435* encodes an *O*-methyl phosphoramidate (MeOPN) transferase adding MeOPN to C2 and C6 of CPS galactose (28). *CJJ81176_1419* encodes a putative methyltransferase involved in CPS biosynthesis (49), and *CJJ81176_1429* is a conserved gene of unknown function. Notably, the *CJJ81176_1429* gene is in the OFF state in the serum-resistant 1211 phasotype. This observation indicates that *CJJ81176_1429* is not required for the PV resistance phenotype in this context. This further suggests that the functional contribution of *CJJ81176_1429* may be compensated by other ON-state PVGs within this phasotype. In addition to phasotypes that increased in frequency during screening, we also examined gene-level ON/OFF dynamics across selection assays (Table 3). Several PVGs remained consistently in the ON state at high ratios throughout the experiments, suggesting a stable contribution to fitness under the tested conditions. Conversely, some loci exhibited transient decreases in ON-state proportion during early selection steps, followed by recovery in later rounds. These patterns indicate that selection does not act uniformly across all loci but instead reflects differential stability and context-dependent contributions of individual PVGs.

**TABLE 3:** Phasevariome of human serum-resistant variants from PLL_81176

PVG	ON/OFF ratio of PVGs			
	Input	Output		
		1st	2nd	3rd
*CJJ81176_0086*	0.25	0.17	0.12	0.10
*CJJ81176_0206*	0.32	0.32	0.35	0.42
*CJJ81176_0646*	0.31	0.39	0.15	0.09
*CJJ81176_0708*	0.29	0.38	0.12	0.06
*CJJ81176_0758*	0.30	1.44	6.53	7.98
*CJJ81176_1160*	0.06	0.17	0.12	0.06
*CJJ81176_1312*	0.23	0.22	0.10	0.09
*CJJ81176_1325*	24.13	8.47	15.79	23.63
*CJJ81176_1327*	0.19	0.38	0.09	0.08
*CJJ81176_1341*	27.15	7.72	10.94	22.63
*CJJ81176_1419*	0.06	4.47	24.62	28.04
*CJJ81176_1421*	0.08	3.21	4.33	2.73
*CJJ81176_1429*	7.01	0.46	0.34	0.41
*CJJ81176_1432*	0.36	0.82	1.43	1.49
*CJJ81176_1435*	0.13	1.92	10.56	56.50
Serum resistance rate ± SD (%)^*a*^	NA	11.0 ± 2.6	122.3 ± 19.1	120.3 ± 7.8

^*a*^ Values indicate the serum resistance rate ± standard deviation (SD). The serum resistance rate (%) was calculated as: (number of surviving output bacteria after serum exposure ÷ number of input bacteria before exposure) × 100. The assay was performed in triplicate. NA indicates not applicable.

**TABLE 4:** Identification of phasotypes and causative genes involved in serum resistance in *C. jejuni* strain 81-176

PVG	Phasotype^*a*^									
	#1	#2	#3	#4	#5	#3v1	#3v2	#3v3	#3v4	#3v5
*CJJ81176_0086*	0	0	0	0	0	0	0	0	0	0
*CJJ81176_0206*	0	0	1	1	0	0	0	0	0	0
*CJJ81176_0646*	1	1	0	0	0	0	0	0	0	0
*CJJ81176_0708*	0	0	0	0	0	0	0	0	0	0
*CJJ81176_0758*	0	1	1	1	1	0	0	0	0	0
*CJJ81176_1160*	0	0	0	0	0	0	0	0	0	0
*CJJ81176_1312*	0	0	0	0	0	0	0	0	0	0
*CJJ81176_1325*	1	1	1	1	1	0	0	0	0	0
*CJJ81176_1327*	0	0	0	0	0	0	0	0	0	0
*CJJ81176_1341*	1	1	1	1	1	0	0	0	0	0
*CJJ81176_1419*	0	1	1	1	1	1	0	1	1	0
*CJJ81176_1421*	0	1	0	0	1	0	0	0	0	0
*CJJ81176_1429*	1	0	1	1	0	1	1	0	1	0
*CJJ81176_1432*	1	1	0	0	1	0	0	0	0	0
*CJJ81176_1435*	1	1	1	1	1	1	1	1	0	0
	4,263	1,211	9,397	9,397	1,211	21	5	17	20	0
Serum resistance rate ± SD (%)^*b*^	31 ± 4	78 ± 5	103 ± 16	114 ± 8	85 ± 16	96 ± 11	ND	ND	ND	ND

^*a*^ Five single colonies (#1 to #5) were isolated from the output of the third exposure, and their genome sequences were analyzed to determine their phasotypes and to conduct serum resistance assays. The phase status of each gene is represented in binary format, with ON as 1 and OFF as 0. Additionally, the phasotypes were converted from binary to decimal format as shown in the bottom row. For example, converting the binary 001000010100111 to decimal gives 4,263. Based on the phasotype 9,397 (#3), various mutant strains (#3v1 to #3v5) were constructed and subjected to serum resistance testing. The following strains were used: #3, SYC2-SV1; #3v1, SYC2-SV2; #3v2, SYC2-SV3; #3v3, SYC2-SV4; #3v4, SYC2-SV5; and #3v5, SYC2-SV6.

^*b*^ ND indicates that surviving output colonies were not detected.

### Systematic phasotype screening identifies phasotypes associated with colonization in IL‑10 knockout mice

*In vitro* results showed PV-GenShift enriches serum-resistant variants and identifies PVGs contributing to immune resistance. To explore whether specific phasotypes are selectively enriched during host colonization, we applied PV-GenShift in an *in vivo* setting using the PLL_81176M library.

Previous studies have shown that C57BL/6 IL-10^+/+^ mice are suitable for colonization studies, while IL-10⁻^/^⁻ mice can serve as a model for both colonization and enteritis (59). In a preliminary experiment, we administered PLL_81176M (2.8 × 10⁹ CFU) or its parent strain 81-176 (1.0 × 10^10^ CFU) to three IL-10^+/+^ mice. After 7 days, no bacterial colonies were detected in fecal samples, indicating a failure to colonize under non-inflammatory conditions. In contrast, IL-10⁻^/^⁻ mice infected with 81-176 showed successful colonization, with fecal bacterial loads ranging from 2.7 × 10⁴ to 6.8 × 10⁶ CFU/g.

Based on these observations, we conducted PV-GenShift screening using PLL_81176M in IL-10⁻^/^⁻ mice to investigate the *in vivo* relevance of specific phasotypes under inflammatory conditions. Briefly, a suspension of 3.0 × 10⁸ CFU was administered to three IL-10⁻^/^⁻ mice. Seven days post-infection, clinical symptoms were assessed, and fecal and cecal samples were collected. Bacterial loads ranged from 1.1 × 10⁸ to 6.7 × 10⁸ CFU/g in the feces and from 9.0 × 10⁸ to 1.1 × 10⁹ CFU/g in the cecal contents, confirming successful colonization (Table 5). Macroscopic examination of the colons from IL-10⁻^/^⁻ mice revealed mucosal erythema, edema, and marked wall thickening. However, it remains unclear whether these changes were directly caused by infection or were a consequence of IL-10 deficiency.

**TABLE 5:** Phasevariome of PLL_81176-derived colonizing variants in the cecum and feces of IL-10 knockout mice

PVG	ON/OFF ratio of PVGs						
	Input	Output^*a*^					
		m#1		m#2		m#3	
		Cecum	Feces	Cecum	Feces	Cecum	Feces
*CJJ81176_0086*	0.29	0.00	0.00	0.00	0.00	0.00	0.00
*CJJ81176_0206*	0.46	0.01	0.01	0.01	0.01	0.09	0.04
*CJJ81176_0646*	0.53	0.00	0.01	0.00	0.01	0.01	0.01
*CJJ81176_0708*	0.71	0.01	0.00	0.00	0.00	0.06	0.03
*CJJ81176_0758*	0.43	0.00	0.00	0.00	0.00	0.00	0.00
*CJJ81176_1160*	0.42	0.00	0.01	0.00	0.01	0.01	0.02
*CJJ81176_1312*	0.45	268.50	690	620.00	1097	12.61	42.5
*CJJ81176_1325*	0.69	0.00	0.00	0.00	0.00	0.00	0.00
*CJJ81176_1327*	0.67	0.00	0.00	0.00	0.01	0.01	0.01
*CJJ81176_1341*	0.35	0.00	0.00	0.01	0.00	0.00	0.01
*CJJ81176_1419*	0.77	321.75	195	639.50	1166	13.19	43.69
*CJJ81176_1421*	0.49	67.67	116	104.00	224	21.75	51.5
*CJJ81176_1429*	2.26	205.40	304.5	189.00	283.75	130.00	55.7
*CJJ81176_1432*	0.94	0.00	0.00	0.00	0.00	0.00	0.01
*CJJ81176_1435*	0.19	0.00	0.00	0.00	0.02	0.00	0.01
Number of bacteria (CFUs/g)	NA	1 × 10^9^	6.7 × 10^8^	9 × 10^8^	1.1 × 10^8^	1.1 × 10^9^	2.2 × 10^8^

^*a*^ Results of phasevariome analysis and colony recovery from the cecum contents and feces of three individual mice are shown.

To assess phasotype selection during colonization, phasevariome analysis was performed by WGS on pooled bacterial populations recovered from the output samples and compared with the input inoculum. For phasevariome analysis of the mouse colonization experiments, gDNA was extracted from pooled bacterial populations corresponding to approximately 20,000 colonies in the input inoculum and approximately 5,000 colonies recovered from the output population per mouse. Four genes—*CJJ81176_1312*, *CJJ81176_1419*, *CJJ81176_1421*, and *CJJ81176_1429*—were enriched in the ON phase, while the remaining PVGs were predominantly in the OFF phase (Table 5; Table S9).

To further characterize the selected phasotype(s), single-colony phasotyping was performed on colonies derived from pooled output populations recovered from the ceca of three infected mice (Table S10). Five colonies were randomly isolated from this pooled population, and their genome sequences were analyzed to determine their phasotypes. All colonies corresponded to the same phasotype, PT284. PT284 is defined by ON-state expression of *CJJ81176_1312*, *CJJ81176_1419*, *CJJ81176_1421*, and *CJJ81176_1429*, with all remaining PVGs in the OFF state, consistent with the pooled phasevariome profile (Table S10).

To assess the composition of the starting library, single-colony phasotyping was also performed on 24 randomly sampled variants prior to infection (Fig. S3). This analysis demonstrated that the input library was not dominated by single-gene ON variants but instead comprised a diverse set of intermediate- to high-order multi-gene phasotypes. However, this sampling was not sufficient to quantitatively determine the initial abundance of PT284 or to exclude underrepresentation or absence of permissive sub-combinations (e.g., configurations lacking one of the four ON genes). Consequently, while PT284 was reproducibly enriched *in vivo*, the possibility that incomplete representation of alternative gene combinations influenced the apparent requirement for this four-gene configuration cannot be fully excluded.

Three of the ON-phase genes—*CJJ81176_1419*, *CJJ81176_1421*, and *CJJ81176_1429*—are located within the CPS biosynthesis cluster. *CJJ81176_1419* and *CJJ81176_1429* have previously been linked to serum resistance (Table 3). *CJJ81176_1421* encodes an additional MeOPN transferase within the CPS biosynthesis cluster, complementing the previously described *CJJ81176_1435* (Table 3), and is involved in the transfer of MeOPN to the C4 position of galactose in the 81-176 CPS (28). The fourth ON-phase gene, *CJJ81176_1312*, is a homolog of *Cj1295* in *C. jejuni* NCTC11168 and is predicted to be involved in the addition of dimethylglyceric acid to *O*-linked pseudaminic acid residues found in flagella (31).

### Systematic phasotype screening reveals heterogeneous phase variation during chicken gut passage

Chickens are a natural reservoir for *C. jejuni* and represent a major source of human campylobacteriosis through contaminated poultry products. To examine PVG expression and host adaptation in a biologically relevant avian host, we applied the PV-GenShift platform to the PLL_81176M library using a chicken colonization model.

A bacterial suspension containing approximately 10⁴ CFU of PLL_81176M (input library) was orally administered to individually housed chickens at 27 days of age. Nineteen days post-inoculation, cecal droppings were collected and plated to recover colonizing bacteria. Bacterial loads ranged from 2.3 × 10⁷ to 6.8 × 10⁷ CFU/g of cecal content (Table 6), confirming successful colonization.

**TABLE 6:** Phasevariome of PLL_81176-derived variants recovered from chicken cecal droppings

PVG	ON/OFF ratio of PVGs			
	Input	Output^*a*^		
		c#1	c#2	c#3
*CJJ81176_0086*	0.32	0.48	222.67	0
*CJJ81176_0206*	0.49	0	140.88	0
*CJJ81176_0646*	0.57	1.94	695	0
*CJJ81176_0708*	0.69	13.67	189.25	0
*CJJ81176_0758*	0.54	0	0	∞
*CJJ81176_1160*	0.37	0.4	190	0
*CJJ81176_1312*	0.51	70	0	0
*CJJ81176_1325*	0.52	1.76	∞	∞
*CJJ81176_1327*	0.69	0.41	0.01	∞
*CJJ81176_1341*	0.33	0	0	0
*CJJ81176_1419*	0.56	0.51	372.67	∞
*CJJ81176_1421*	0.20	0.04	∞	∞
*CJJ81176_1429*	2.18	∞	∞	∞
*CJJ81176_1432*	0.92	1.89	∞	0
*CJJ81176_1435*	0.53	∞	151	0
Number of bacteria in the cecal droppings (CFUs/g)	NA	2.3 × 10^7^	4.2 × 10^7^	6.8 × 10^7^

^*a*^ The results of phasevariome analysis and colony recovery from the cecal droppings of three individual chickens are presented. ∞ indicates that no OFF-state reads were detected for the gene, such that the ON/OFF ratio (ON reads divided by OFF reads) is mathematically infinite.

To assess the impact of chicken gut passage on PVG expression, phasevariome analysis was performed by WGS on the pooled output populations and compared with the input library (Table 6; Table S11). For these analyses, gDNA was extracted from pooled bacterial populations corresponding to approximately 20,000 colonies used to prepare the input library and approximately 6,000 colonies recovered from the output population per individual bird. The chicken inoculum consisted of approximately 10⁴ CFUs, representing the upper bound on the number of phasotypes entering the host. Given the estimated diversity of the starting library, this inoculum size is expected to capture only a fraction of the available phasotype space, likely representing a subset of several hundred to a few thousand phasotypes. Following a single round of host passage, all chickens exhibited marked changes in ON/OFF ratios across multiple PVGs, although the overall patterns varied among individuals.

Despite this inter-individual variability, consistent trends were observed for specific loci. *CJJ81176_1429* reproducibly exhibited increased ON-state representation in all chickens, whereas *CJJ81176_1341* was consistently enriched in the OFF phase. *CJJ81176_1429* has previously been implicated in serum resistance (Table 4) and also showed ON-phase enrichment following mouse colonization (Table 5), suggesting a conserved contribution to host adaptation across distinct host environments. In contrast, *CJJ81176_1341* encodes a hypothetical protein (Table 1), and its consistent OFF-phase enrichment may indicate a selective disadvantage during chicken colonization.

## DISCUSSION

PV is a key adaptive strategy in *C. jejuni*, enabling rapid and reversible alterations of surface structures through stochastic changes in SSRs. This ON/OFF switching facilitates immune evasion and environmental adaptation, but also complicates functional analysis of individual PVGs. While genome sequencing reveals strain-specific gene content, PV introduces an additional layer of phenotypic heterogeneity by generating reversible switching at multiple loci within otherwise clonal populations. Our findings highlight that meaningful interpretation of *C. jejuni* pathogenesis requires explicit consideration of both genomic diversity and PV-driven heterogeneity (60).

To address these challenges, we developed PV-GenShift, a genome-scale screening platform based on a genetically stabilized library of phase-locked variants. By permanently fixing the ON/OFF states of 15 PVGs, PV-GenShift enables reproducible and high-resolution analysis of defined phasotypes under selective pressures. Using this framework, we identified specific phasotypes that enhance serum resistance, providing direct evidence that combinations of PVGs, rather than individual loci acting independently, contribute to host adaptation (Table 4). In contrast, although phasotype enrichment was reproducibly observed during host colonization, definitive functional configurations were not consistently observed across all *in vivo* models.

In murine colonization experiments, specific phasotypes available within the starting phase-locked library were consistently enriched following infection (Table 5). Because phasotypes cannot be inferred from colony morphology, single-colony recovery from the library is necessarily random, and the reproducible enrichment of the same phasotype across independently infected mice argues against purely stochastic loss and instead supports consistent selective enrichment. While population bottlenecks cannot be completely excluded, these observations are more consistent with selection acting on available phasotype configurations *in vivo*. In contrast, chicken colonization experiments revealed divergent phasotypes across individuals, with consistent but non-identical enrichment patterns and no convergence on a single dominant phasotype (Table 6). This variability likely reflects a combination of non-selective bottlenecks and host-specific ecological constraints, which can obscure phasotype-specific selection in avian hosts (49, 61).

Across serum exposure, murine colonization, and chicken passage, PVGs involved in CPS biosynthesis were consistently enriched in the ON phase. In serum resistance assays, ON-state expression of *CJJ81176_1419*, *CJJ81176_1429*, and *CJJ81176_1435* correlated with survival. In mice, *CJJ81176_1419*, *CJJ81176_1421*, and *CJJ81176_1429* defined a dominant phasotype, whereas in chickens, enrichment of *CJJ81176_1429* in the ON state was observed without convergence on a single configuration. These genes encode CPS-modifying enzymes, including MeOPN transferases and predicted methyltransferases: *CJJ81176_1421* adds MeOPN to C4 of galactose, *CJJ81176_1435* modifies C2 and C6 (28), and *CJJ81176_1419* likely encodes a methyltransferase, while *CJJ81176_1429* remains uncharacterized. In strain NCTC11168, combinations of MeOPN decoration and methylation influence serum resistance, phage susceptibility, and antigenic variation (10). Consistent with this, we previously demonstrated that Penner serotype antigenicity is determined by the PVG *cj1426*, reinforcing the functional importance of CPS methylation (55). Loss of MeOPN in strain 81-176 increases complement susceptibility and epithelial invasion (28, 62), supporting a role for these modifications in virulence (28, 39, 56, 59, 63). Together, these observations support a model in which CPS modifications act as a modular system, with different ON/OFF combinations fine-tuning surface architecture under distinct selective pressures. It is important to note that resistance to serum, antimicrobial peptides, or bacteriophages in *C. jejuni* is not universal but is strain- and context-dependent. In particular, the serum resistance observed here reflects selection under the specific experimental conditions used, and different immune factors or host contexts may yield different outcomes.

Comparisons with prior studies further reinforce the biological relevance of the enrichment patterns observed here. In strain NCTC11168, mouse colonization has been associated with ON-phase enrichment of *Cj1295* and *Cj1429*, and OFF-phase enrichment of *Cj0170* and *Cj1306* (27, 50, 51, 64). In the 81-176 background, *CJJ81176_1312* encodes a homolog of Cj1295 involved in flagellar glycosylation and immune evasion (31), while *CJJ81176_0206* and *CJJ81176_1341* are homologous to *Cj0170* and *Cj1306*, respectively. The conservation of these host-specific patterns across strains underscores the utility of PV-GenShift for dissecting biologically meaningful PVG contributions.

### Methodological context, limitations, and future directions

PV-GenShift addresses key limitations of conventional PV studies, which have largely relied on genetically unstable wild-type populations analyzed using colony-based approaches such as Sanger sequencing or fragment analysis (50, 51, 54, 64). While these methods have been instrumental in establishing the prevalence and biological relevance of PV, they are labor-intensive, difficult to scale, and poorly suited for interrogating combinatorial phasotypes involving multiple PVGs. By combining MuGENT-SSR editing with phase locking, PV-GenShift stabilizes genetic context, eliminates spontaneous switching, and enables reproducible functional interrogation of defined PVG combinations. Generation of independently derived libraries may further facilitate broader exploration of phenotypic space.

Several limitations should be considered when interpreting these findings. Although short-read WGS was performed, complete genome sequences were not obtained for every phase-locked variant, and unintended mutations outside the targeted SSR loci cannot be fully excluded. However, because each ON and OFF configuration is represented by multiple independent variants, such background mutations are unlikely to be systematically associated with specific phase states. In addition, the phase-locked library samples only a subset of the theoretical 2¹⁵ phasotype space. Finite library size, locus-specific editing efficiencies, and restricted inoculum sizes further constrain the diversity of phasotypes entering host environments. Consequently, observed enrichment patterns should be interpreted as selection acting on available phasotypes rather than exhaustive coverage of all possible ON/OFF combinations, including potentially functional low-frequency sub-combinations. Future improvements could include optimizing locus-specific editing efficiencies and increasing sampling depth to achieve more balanced representation of ON/OFF states across PVGs

A further practical limitation of PV-GenShift lies in its reliance on natural transformation via MuGENT-SSR. Many *C. jejuni* isolates exhibit limited or unstable competence (65), restricting the direct applicability of this approach across diverse lineages. Extending PV-GenShift to transformation-refractory strains will require alternative genome-engineering strategies, including CRISPR-based editing or recombinase-mediated systems.

Population-level approaches, including fragment-based analyses and mathematical inference models, can provide high-dimensional descriptions of phasotype distributions in wild-type populations (66). In particular, approaches that integrate single-colony and population-level data have been developed to improve estimation of phasotype distributions from limited sampling, and our observations are consistent with such frameworks in indicating the presence of numerous low-frequency phasotypes. These approaches are complementary to PV-GenShift and can be incorporated into a discovery-to-validation framework, whereby candidate phasotypes identified at the population level are reconstructed and functionally tested using phase-locked variants. In parallel, the quantitative framework described in reference 66, when combined with single-colony phasotyping, can also be applied to assess the effective composition and accessibility of phasotype space within a given library itself.

Future extensions of PV-GenShift could include construction of modular libraries targeting defined functional classes of PVGs (e.g., CPS biosynthesis genes, LOS biosynthesis genes, or flagellar modification genes), generation of independently derived libraries to broaden exploration of phasotype space, and application to additional *C. jejuni* lineages and other bacterial pathogens. These strategies would help reduce representation bias and improve access to otherwise underrepresented phasotypes. Expanding the approach to other SSR types (e.g., polyA/T tracts) or non-SSR PV mechanisms would further increase its generality. Together, these developments will be essential for scaling the framework and for dissecting the complex contributions of combinatorial PV to bacterial adaptation.

### Conclusions

In conclusion, our findings demonstrate that SSR-mediated ON/OFF switching of multiple PVGs exerts a combinatorial influence on *C. jejuni* adaptation to immune pressure and host environments. By stabilizing PVG expression states, PV-GenShift enables reproducible and systematic functional interrogation of defined phasotypes, complementing existing population-level approaches. This platform provides a generalizable framework for dissecting PV-driven phenotypic diversity and identifying causative gene combinations underlying virulence, colonization, and immune resistance.

## MATERIALS AND METHODS

### Bacterial culture conditions

*C. jejuni* strains (Table S1) were cultured for 48 h at 37°C on *Brucella* broth (BB; Becton Dickinson and Co.) plates with 1.5% agar (BBA; Kyokuto Pharmaceutical Industrial Co., Ltd.) under microaerophilic conditions using Anaeropack MicroAero (Mitsubishi Gas Chemical Company, Inc.). Motility was assessed on BB plates with 0.3% agar (BBAM). For liquid culture, single colonies from BBA plates were inoculated into 5 mL brain heart infusion broth (BHB; Becton Dickinson and Co.) in 25 mL tubes and incubated overnight at 37°C with shaking (160 rpm) using a Precyto MG-71M-A system (Taitec Corporation), which maintained 5% O_2_, 10% CO_2_, and 85% N_2_ at 10 mL/min. Stocks were preserved at –80°C in BHB with 20% glycerol (BHBS). Antibiotics were added as needed: 25 μg/mL chloramphenicol, 50 μg/mL kanamycin.

### DNA manipulation

PCR was performed using a LifeECO thermal cycler (version 1.04; Bioer Technology Japan Co., Ltd.) with Quick Taq HS DyeMix or KOD One PCR Master Mix (both from Toyobo Co., Ltd.). Sanger sequencing was outsourced to Fasmac Co., Ltd. PCR products were purified using a High Pure PCR Product Purification Kit (Roche Diagnostics K.K.). Primers (Table S2) were obtained from Fasmac Co., Ltd. and Hokkaido System Science Co., Ltd. Genomic and plasmid DNA were extracted using a DNeasy Blood and Tissue Kit (Qiagen K.K.) and a High Pure Plasmid Isolation Kit (Roche Diagnostics K.K.), respectively.

### Natural transformation of *C. jejuni* cells using methylated DNA

Natural transformation using methylated donor DNA followed a published protocol (55). Donor DNA fragments were PCR-amplified (Table S2), methylated *in vitro*, introduced into *C. jejuni*, and transformants were selected on BBA with antibiotics. Template DNA and primer combinations are listed in Table S3.

### WGS

WGS was outsourced to Rhelixa, Inc. and performed on an Illumina NovaSeq 6000 using the NEBNext Ultra II DNA Library Prep Kit (New England Biolabs Inc.). Sequencing was 150 bp paired-end, generating ~1 GB per sample.

### MuGENT-SSR

Phase-locked strains were generated using MuGENT-SSR with methylated donor DNA (55). Selected DNA (e.g., Δ*flaA::kan* or Δ*flaA::cat*; Table S3) introduced antibiotic resistance at *flaA*, while unselected DNA introduced locked ON/OFF mutations into 15 PVGs (Table S3). After transformation, colonies were selected on BBA with antibiotics, cultured in 96-well plates, and screened by MASC PCR using primer mixes (Mix ON1–3 or OFF1–3; Table S5). PCR conditions were as follows: 94°C for 5 min; 35 cycles of 94°C for 30 s, 55.2°C for 30 s, and 68°C for 2 min. Positive clones were re-isolated and confirmed.

### Construction of a phase-locked variant library

A phase-locked variant library was constructed using MuGENT-SSR. All serial rounds of MuGENT-SSR were performed sequentially using a single parental culture.

Each round of transformation was carried out on the population obtained from the preceding round, rather than on independently derived cultures, in order to generate a controlled baseline phase-locked library. The SYC2-0 strain, in which all 15 PVGs were locked in the OFF phase (see Table S1 and Fig. S2 for construction), was cultured in BHB until the A₆₀₀ reached 0.15. One milliliter of this culture was mixed with 0.5 μg of selected DNA (Δ*flaA::cat*) and 2 μg of each unselected DNA carrying locked-ON mutations, then incubated at 37°C for 18 h. The resulting chloramphenicol-resistant transformants (~20,000 CFUs) were suspended in 10 mL of BHBS, and genomic DNA was extracted from 1 mL of this suspension.

The remaining suspension was diluted in 5 mL of BHB to an A₆₀₀ of 0.05 and cultured until the A₆₀₀ reached 0.15. A second round of MuGENT-SSR was then performed using selected DNA with a different antibiotic resistance marker (Δ*flaA::kan*) and the same unselected DNA. This process was repeated for five cycles, each using a different antibiotic resistance marker. gDNA was extracted from the resulting kanamycin-resistant transformants and used for subsequent rounds of MuGENT-SSR.

WGS was performed on the extracted DNA, and the number of reads corresponding to locked-ON and locked-OFF mutations in each PVG was quantified using an in-house BLAST+-based tool (67), named “PVfinder_81176.” After trimming raw FASTQ reads with Sickle (68), the reads with 100% identity to either the ON/OFF phase sequences (Table S6) were counted. For each PVG, the ON/OFF ratio was calculated by dividing the number of ON reads by the number of OFF reads. A ratio close to 1 indicates a balanced population (high diversity), while ratios approaching 0 or infinity indicate dominance of OFF or ON variants, respectively. A population with ON/OFF ratios near 1 for all PVGs was selected as a phase-locked library candidate (PLL_81176). Short-read WGS was performed to assess editing outcomes and to analyze phasevariome profiles. However, complete genome sequences were not generated for every individual phase-locked variant, and therefore additional point mutations outside the targeted SSR loci may be present in some variants and were not comprehensively cataloged.

### Restoration of motility in phase-locked variants

Since the *flaA* gene was disrupted by the insertion of an antibiotic resistance cassette during MuGENT-SSR, the resulting phase-locked variants lost motility, a key factor in *C. jejuni* pathogenicity (36, 37, 40, 69). To restore motility, the wild-type *flaA^+^* gene was reintroduced via natural transformation. Approximately 20,000 phase-locked variant colonies from PLL_81176 were suspended in 10 mL of BHB, diluted to an A_600_ of 0.05 in 5 mL of fresh BHB, and cultured until the A_600_ reached 0.15. Transformation was then performed using *flaA^+^* DNA (Table S3). Briefly, a 50 μL aliquot of the transformation mixture was spotted onto BBAM plates and incubated at 37°C for 24–48 h. Agar plugs containing motile colonies were then transferred to 5 mL of BHB in a six-well plate and incubated at 37°C for 24 h. After centrifugation, the bacterial pellet was resuspended in BHBS to an A_600_ of 0.5. Aliquots (100 μL) of this suspension were stored at –80°C as the final phase-locked variant library, designated PLL_81176M.

### Serum resistance assays

Serum resistance assays were performed with slight modifications based on the method described by Maue et al. (39). Briefly, 1 mL of an overnight *C. jejuni* culture in 5 mL of BHB was washed twice with 1 mL of minimal essential medium (MEM; Nacalai Tesque, Inc.) and adjusted to an A_600_ of 0.1 in MEM (defined as the initial input bacteria). A 100 μL aliquot of a 1:10,000 dilution of this suspension, containing approximately 10,000 CFUs, was added to each well of a 48-well plate containing 900 μL of prewarmed MEM supplemented with 11.25% human complement serum (Sigma-Aldrich). The plate was incubated under microaerobic conditions at 37°C for 60 min. The percentage of surviving output bacteria after 60 min relative to the initial input was determined via serial dilution and plating on mCCDA selective agar (Kanto Chemical Co., Inc.).

### PV-GenShift of human serum-resistant phase variants from PLL_81176M

To select serum-resistant phase variants, the PLL_81176M library was serially passaged in human complement serum for three rounds. After each passage, surviving bacteria were collected and used as input for the subsequent round. gDNA was extracted from the initial input population and from surviving output populations at each passage. For output samples, bacteria recovered from three independent replicates were pooled and used both as inoculum for the next passage and as the source material for gDNA extraction prior to WGS.

For phasevariome analysis, gDNA was extracted from pooled bacterial populations at defined stages of selection. In the first round, gDNA was extracted from approximately 10,000 colonies derived from the pre-exposure inoculum and from approximately 3,000 colonies recovered after serum exposure (60 min). In all subsequent rounds, gDNA was extracted solely from post-exposure output populations, with approximately 5,000 colonies per round. Thus, the number of colonies contributing to gDNA extraction defines the effective population size and the upper bound on observable phasotype diversity in the sequencing analysis. ON/OFF ratios of all PVGs were calculated using the PVfinder_81176 tool.

### PV-GenShift of mouse intestinal colonized phase variants from PLL_81176M

Mouse colonization experiments were conducted with minor modifications to the protocol described by Mansfield et al. (59). *Campylobacter*-free C57BL/6 IL-10^+/+^ and congenic IL-10⁻^/^⁻ mice were maintained under specific pathogen-free conditions at Kyorin University. The PLL_81176M library was cultured on blood agar plates to generate approximately 20,000 colonies, which were suspended in PBS to prepare the input inoculum and extract gDNA. Each mouse (*n* = 3) was orally inoculated with 0.2 mL of the suspension (3.0 × 10⁸ CFU).

Seven days post-infection, fecal and cecal samples were collected, suspended in PBS, and plated on mCCDA agar. WGS was performed on both input and output samples to determine ON/OFF ratios of all PVGs. For phasevariome analysis, gDNA was extracted from pooled populations corresponding to approximately 20,000 colonies in the input inoculum and approximately 5,000 colonies recovered per mouse, defining the effective population size and observable phasotype diversity. Wild-type and IL-10-deficient mice were also infected with the parental strain 81-176 (1.0 × 10¹⁰ CFU) as a control.

### PV-GenShift of chicken intestinal colonized phase variants from PLL_81176M

Chicken colonization experiments were performed with minor modifications of the method described by Jones et al. (70). The PLL_81176M library was cultured on BBA plates, and approximately 20,000 colonies were suspended in PBS to prepare the input population and extract gDNA. From this suspension, ~10⁴ CFU were orally administered to individually housed, *Campylobacter*-free Hy-Line Brown chickens (*n* = 3). As each CFU corresponds to a single colony, this value represents the absolute upper bound on the number of phasotypes entering the host.

Nineteen days post-inoculation, cecal droppings were collected, suspended in PBS, and plated on BBA supplemented with CCDA selective supplement (Kanto Chemical Co., Inc.). WGS was performed on both input and output samples to calculate ON/OFF ratios of all PVGs. For phasevariome analysis, gDNA was extracted from pooled populations corresponding to approximately 20,000 colonies in the input inoculum and approximately 5,000 colonies recovered per bird, defining the effective population size and upper bound on observable phasotype diversity.

## AFTER EPUB

*[This article was published on 10 June 2026 with an error in the affiliation for Akiko Kubomura. The affiliation was corrected in the current version, posted on 15 June 2026.]*

## SUPPLEMENTAL MATERIAL

Supplemental material msphere.00064-26-s0001.docx Fig. S1 to S4; Tables S1 to S11.

ASM does not own the copyrights to Supplemental Material that may be linked to, or accessed through, an article. The authors have granted ASM a non-exclusive, world-wide license to publish the Supplemental Material files. Please contact the corresponding author directly for reuse.
