# Supplementary material for: Genome-scale dissection of phase-variable gene function in Campylobacter jejuni using a stabilized phasotype library
Source: mSphere. 2026 Jun 10;11(6):e00064-26. doi: 10.1128/msphere.00064-26 (PMC13317227; doi:10.1128/msphere.00064-26)
Supplement: Supplemental material — Fig. S1 to S4; Tables S1 to S11. [file msphere.00064-26-s0001.docx]

**Supplemental Materials for**

Genome-scale dissection of phase-variable gene function in *Campylobacter jejuni* using a stabilized phasotype library

Shouji Yamamoto*, Ken-ichi Lee, Akiko Kubomura, Sunao Iyoda, Yukihiro Akeda, Takaaki Shimohata, Chihiro Aikawa, Masashi Okamura, Fuhito Hojo, Takako Osaki, and Jiro Mitobe

*Shouji Yamamoto

**Email:**  [yamamoto.sh@jihs.go.jp](mailto:yamamoto.sh@jihs.go.jp)

**This PDF file includes:**

Figure S1 to S4

Tables S1 to S11

**Figures**

**Fig. S1**

**Fig. S1. Genomic organization and reading frames of *CJJ81176_1421* and *CJJ81176_1420*.**This figure illustrates the relative genomic positions and reading frames of the adjacent genes *CJJ81176_1421* and *CJJ81176_1420* in *C. jejuni* strain 81-176. The polyG tract (highlighted in red) varies in length (e.g., 9 or 10 G residues), resulting in a frameshift that alters the downstream coding sequence. The start and stop codons for both genes are indicated. Notably, a clear Shine–Dalgarno (SD) sequence is present upstream of *CJJ81176_1421*, but no such sequence is evident for *CJJ81176_1420*. Given the shared reading frame and the absence of an independent SD sequence for *CJJ81176_1420*, this gene is likely a misannotation. These observations suggest that this region represents a single PVG, *CJJ81176_1421*, whose expression is regulated by changes in the length of the SSR tract.

**Fig. S2**

A

B

**Fig. S2. Construction of the SYC2-0K strain, in which all 15 PVGs are locked in the OFF phase.**

(A) The SYC2‑0K strain was constructed using MuGENT‑SSR with methylated donor DNA. In each MuGENT‑SSR round, selected DNA carrying an antibiotic resistance marker inserted at the *flaA* locus (e.g., Δ*flaA*::*kan* or Δ*flaA*::*cat*) was co‑transformed to enable selection of successful recombinants, while unselected DNA fragments carried locked‑OFF alleles of phase‑variable genes. Rather than introducing all 15 mutations simultaneously, phase‑variable genes were divided into three groups corresponding to the MASC PCR primer mixes (Mix OFF1, Mix OFF2, and Mix OFF3, Table S5), and MuGENT‑SSR was performed sequentially for each group. After each round, 40-96 colonies were screened by MASC PCR to confirm OFF‑phase locking of the targeted gene set before proceeding to the next group. Completion of these sequential steps resulted in all 15 phase‑variable genes being stably locked in the OFF phase.

(B) Successful construction of the SYC2‑0K strain was verified by MASC PCR. OFF‑phase primer mixes (Mix OFF1–3) were used to confirm OFF states at all 15 loci, and ON‑phase primer mixes (Mix ON1–3; Table S5) were used to confirm the absence of ON‑phase alleles. As a control, the parental strain 81‑176 was analyzed in parallel using the same primer sets. Because allele‑specific MASC PCR primers selectively amplify only the locked ON or OFF alleles, no PCR products were detected for the parental strain, which contains heterogeneous and unlocked SSR configurations. This result confirms the specificity of the MASC PCR system for detecting phase‑locked alleles.

**Fig. S3**

A

B

C

**Fig. S3. Single‑colony analysis of 24 representative phase‑locked library variants randomly sampled from the library.**

**(A)** Twenty‑four colonies were randomly selected from the phase‑locked library PLL_81176M. Phasotypes were determined by MASC‑PCR using ON‑phase primer mixes (Mix ON1–3).

**(B)** Summary of the MASC‑PCR results shown in panel A.

**(C)** Phasotyping of the 24 variants. Phase states are ordered by gene number and displayed in binary notation (ON = 1, OFF = 0). The numerical value shown at the bottom represents the corresponding decimal representation of each phasotype.

**Fig. S4**

**Fig. S4. Inferred distribution of ON‑state gene counts per colony in the initial phase‑locked library.**
The histogram shows the inferred distribution of the number of phase‑variable genes in the ON state per colony in the initial phase‑locked library PLL_81176M (~20,000 colonies). The distribution was derived from single‑colony phasotyping data (Fig. S3) and should therefore be interpreted as an approximate representation of the library composition.

Consistent with these data, the library is enriched in intermediate‑ to high‑order phasotypes, with most colonies carrying approximately five to nine phase‑variable genes in the ON state, and is not dominated by single‑gene ON configurations.

.

Tables

**Table S1.** Strains and plasmids used in this study.

| Strain or plasmid | Characteristics | Source or reference |
| --- | --- | --- |
| Strain |  |  |
| 81-176 | *C. jejuni* raw milk origin, serotype R (antigenic factors HS23/HS36) | (1) |
| SYC2-0K | 81-176 Δ*flaA*::*kan CJJ81176_0086*^OFF^ *CJJ81176_0206* ^OFF^ *CJJ81176_0646* ^OFF^ *CJJ81176_0708* ^OFF^ *CJJ81176_0758* ^OFF^ *CJJ81176_1160* ^OFF^ *CJJ81176_1312* ^OFF^ *CJJ81176_1325* ^OFF^ *CJJ81176_1327* ^OFF^ *CJJ81176_1341* ^OFF^ *CJJ81176_1419* ^OFF^ *CJJ81176_1421* ^OFF^ *CJJ81176_1429* ^OFF^ *CJJ81176_1432* ^OFF^ *CJJ81176_1435* ^OFF^ (PT0) | This study |
| SYC2-0 | SYC2-0K *flaA*^+^ | This study |
| SYC2-SV1 | A PLL_81176M variant resistant to human complement serum *CJJ81176_0086*^OFF^ *CJJ81176_0206* ^ON^ *CJJ81176_0646* ^OFF^ *CJJ81176_0708* ^OFF^ *CJJ81176_0758* ^ON^ *CJJ81176_1160* ^OFF^ *CJJ81176_1312* ^OFF^ *CJJ81176_1325* ^ON^ *CJJ81176_1327* ^OFF^ *CJJ81176_1341* ^ON^ *CJJ81176_1419* ^ON^ *CJJ81176_1421* ^OFF^ *CJJ81176_1429* ^ON^ *CJJ81176_1432* ^OFF^ *CJJ81176_1435* ^ON^ (PT9397) | This study |
| SYC2-SV2C | SYC2-SV1 Δ*flaA*::*cat CJJ81176_0086*^OFF^ *CJJ81176_0206* ^OFF^ *CJJ81176_0646* ^OFF^ *CJJ81176_0708* ^OFF^ *CJJ81176_0758* ^OFF^ *CJJ81176_1160* ^OFF^ *CJJ81176_1312* ^OFF^ *CJJ81176_1325* ^OFF^ *CJJ81176_1327* ^OFF^ *CJJ81176_1341* ^OFF^ *CJJ81176_1419* ^ON^ *CJJ81176_1421* ^OFF^ *CJJ81176_1429* ^ON^ *CJJ81176_1432* ^OFF^ *CJJ81176_1435* ^ON^ (PT21) | This study |
| SYC2-SV2 | SYC2-SV2C *flaA*^+^ | This study |
| SYC2-SV3 | SYC2-SV2C *flaA*^+^ *CJJ81176_0086*^OFF^ *CJJ81176_0206* ^OFF^ *CJJ81176_0646* ^OFF^ *CJJ81176_0708* ^OFF^ *CJJ81176_0758* ^OFF^ *CJJ81176_1160* ^OFF^ *CJJ81176_1312* ^OFF^ *CJJ81176_1325* ^OFF^ *CJJ81176_1327* ^OFF^ *CJJ81176_1341* ^OFF^ *CJJ81176_1419* ^OFF^ *CJJ81176_1421* ^OFF^ *CJJ81176_1429* ^ON^ *CJJ81176_1432* ^OFF^ *CJJ81176_1435* ^ON^ (PT5) | This study |
| SYC2-SV4 | SYC2-SV2C *flaA*^+^ *CJJ81176_0086*^OFF^ *CJJ81176_0206* ^OFF^ *CJJ81176_0646* ^OFF^ *CJJ81176_0708* ^OFF^ *CJJ81176_0758* ^OFF^ *CJJ81176_1160* ^OFF^ *CJJ81176_1312* ^OFF^ *CJJ81176_1325* ^OFF^ *CJJ81176_1327* ^OFF^ *CJJ81176_1341* ^OFF^ *CJJ81176_1419* ^ON^ *CJJ81176_1421* ^OFF^ *CJJ81176_1429* ^OFF^ *CJJ81176_1432* ^OFF^ *CJJ81176_1435* ^ON^ (PT17) | This study |
| SYC2-SV5 | SYC2-SV2C *flaA*^+^ *CJJ81176_0086*^OFF^ *CJJ81176_0206* ^OFF^ *CJJ81176_0646* ^OFF^ *CJJ81176_0708* ^OFF^ *CJJ81176_0758* ^OFF^ *CJJ81176_1160* ^OFF^ *CJJ81176_1312* ^OFF^ *CJJ81176_1325* ^OFF^ *CJJ81176_1327* ^OFF^ *CJJ81176_1341* ^OFF^ *CJJ81176_1419* ^ON^ *CJJ81176_1421* ^OFF^ *CJJ81176_1429* ^ON^ *CJJ81176_1432* ^OFF^ *CJJ81176_1435* ^OFF^ (PT20) | This study |
| SYC2-SV6 | SYC2-SV2C *flaA*^+^ *CJJ81176_0086*^OFF^ *CJJ81176_0206* ^OFF^ *CJJ81176_0646* ^OFF^ *CJJ81176_0708* ^OFF^ *CJJ81176_0758* ^OFF^ *CJJ81176_1160* ^OFF^ *CJJ81176_1312* ^OFF^ *CJJ81176_1325* ^OFF^ *CJJ81176_1327* ^OFF^ *CJJ81176_1341* ^OFF^ *CJJ81176_1419* ^OFF^ *CJJ81176_1421* ^OFF^ *CJJ81176_1429* ^OFF^ *CJJ81176_1432* ^OFF^ *CJJ81176_1435* ^OFF^ (PT0) | This study |
| SYC2005 | 81-176 Δ*kpsE*::*kan* | This study |
| Plasmid |  |  |
| pSYC-*cat* | pUCFa *cat* from *C. coli* | (2) |
| pSYC-*kan* | pUCFa *kan* from *C. coli* | (2) |

**Table S2.** Primers used in this study.

| Primer name | Sequence (5′ to 3′ direction)* |
| --- | --- |
| 176_1439-f1E | GGGGAATTCTACCTGGTTAACTCCTCGTC |
| 176_1439-kan-r1 | AATGGTTCGCTGGGTTTATCCTTGGTGCTG CAATCAATGT |
| kan-176_1439-f1 | CCTAGATTTAGATGTCTAAAAAGCGTCCAGATATTCCAGAAAGC |
| 176_1439-r1E | GGGGAATTCCTCAGGGTGAAATTCTACCTC |
| 6_0086-f1E | GGGGAATTCGAGTCGTGAAATGGTGATTTTGTAG |
| 6_0086-ON-f1 | GAAGTGCATTTAACTTGGGGCGGAGTAATAGGCTTTAGGG |
| 6_0086-ON-r1 | CCCTAAAGCCTATTACTCCGCCCCAAGTTAAATGCACTTC |
| 6_0086-OFF(-1)-f1 | GAAGTGCATTTAACTTGGGGAGGCTAATAGGCTTTAGGG |
| 6_0086-OFF(-1)-r1 | CCCTAAAGCCTATTAGCCTCCCCAAGTTAAATGCACTTC |
| 6_0086-MASCR1 | GCGGAGAGAGAAAATAAAGCATC |
| 6_0086-MASCmF2 | GAAGTGCATTTAACTTGGGGAGCC |
| 6_0086-MASCmF1 | GAAGTGCATTTAACTTGGGGCGCA |
| 6_0086-r1E | GGGGAATTCGTCTAGCTTTTGTGATCTTCCC |
| 6_0206-f1E | GGGGAATTCGAGCAATGATGCATAAAATGAAGGTG |
| 6_0206-ON-f1 | CTAAGTATTTTAAAAATATAACCGGCGGAGGTATAGAGCCTTATGGC |
| 6_0206-ON-r1 | GCCATAAGGCTCTATACCTCCGCCGGTTATATTTTTAAAATACTTAG |
| 6_0206-OFF(-1)-f1 | CTAAGTATTTTAAAAATATAACCGGAGGCGTATAGAGCCTTATGGC |
| 6_0206-OFF(-1)-r1 | GCCATAAGGCTCTATACGCCTCCGGTTATATTTTTAAAATACTTAG |
| 6_0206-MASCR1 | ACTATAGCCTTTATCGCTCATCATG |
| 6_0206-MASCmF2 | CTAAGTATTTTAAAAATATAACCGGAGCC |
| 6_0206-MASCmF1 | CTAAGTATTTTAAAAATATAACCGGCGCA |
| 6_0206-r1E | GGGGAATTCGAGCAATGATGCATAAAATGAAGGTG |
| 6_0646-f1E | GGGGAATTCGACTCAAAATCTCCTGAAAATTCAGG |
| 6_0646-ON-f1 | CCATTTAAACTAATGAGGGGCGGAGGTATTAGAACGATTTTG |
| 6_0646-ON-r1 | CAAAATCGTTCTAATACCTCCGCCCCTCATTAGTTTAAATGG |
| 6_0646-OFF(-1)-f1 | CCATTTAAACTAATGAGGGGAGGCGTATTAGAACGATTTTG |
| 6_0646-OFF(-1)-r1 | CAAAATCGTTCTAATACGCCTCCCCTCATTAGTTTAAATGG |
| 6_0646-MASCR1 | CATATGTTCCATGATATCTAGTAAAATCG |
| 6_0646-MASCmF2 | CCATTTAAACTAATGAGGGGAGCC |
| 6_0646-MASCmF1 | CCATTTAAACTAATGAGGGGCGCA |
| 6_0646-r1E | GGGGAATTCGACTCAAAATCTCCTGAAAATTCAGG |
| 6_0708-f1E | GGGGAATTCAACCTCATCTTCAACTTCGGC |
| 6_0708-ON-f1 | CTTGCTATAAATTTTAATTTTACCCCACCGCATAAAGATAAATTAG |
| 6_0708-ON-r1 | CTAATTTATCTTTATGCGGTGGGGTAAAATTAAAATTTATAGCAAG |
| 6_0708-OFF(-1)-f1 | CTTGCTATAAATTTTAATTTTACCCCGCCAATAAAGATAAATTAG |
| 6_0708-OFF(-1)-r1 | CTAATTTATCTTTATTGGCGGGGTAAAATTAAAATTTATAGCAAG |
| 6_0708-MASCR1 | CATCATTTTTGATTCTGTTTCATCTATGG |
| 6_0708-MASCmF2 | CTTGCTATAAATTTTAATTTTACCCCGCGA |
| 6_0708-MASCmF1 | CTTGCTATAAATTTTAATTTTACCCCACGG |
| 6_0708-r1E | GGGGAATTCTGACACAGGTAGTGGTAAGAC |
| 6_0758-f1E | GGGGAATTCGAAAGCATAGCCATAAAATGCG |
| 6_0758-ON-f1 | CGTTTACTGACAGGCGGGGCGGAGATTTAACAATCAAACC |
| 6_0758-ON-r1 | GGTTTGATTGTTAAATCTCCGCCCCGCCTGTCAGTAAACG |
| 6_0758-OFF(-1)-f1 | CGTTTACTGACAGGCGGGGAGGCATTTAACAATCAAACC |
| 6_0758-OFF(-1)-r1 | GGTTTGATTGTTAAATGCCTCCCCGCCTGTCAGTAAACG |
| 6-0758_MASC-F1 | CTTCTTCGGTTTGTTTTGCG |
| 6-0758_MASC_ON-R1 | AATGGTTTGATTGTTAAATCTCGG |
| 6_0758-MASCR1 | GTAATGTTCTTCCACCGTAAATTTCTCC |
| 6_0758-MASCmF2 | CGTTTACTGACAGGCGGGGAGCC |
| 6_0758-r1E | GGGGGAATTCGCATCATCTGAAACTTCAAAGC |
| 6_1312-f1E | GGGGAATTCGCATGCGTTATTTTATAGGGG |
| 6_1312-ON-f1 | GAAATTTTAAATAAAACTCTGGGCGGAGGTATACTCAAATTTCACTC |
| 6_1312-ON-r1 | GAGTGAAATTTGAGTATACCTCCGCCCAGAGTTTTATTTAAAATTTC |
| 6_1312-OFF(-1)-f1 | GAAATTTTAAATAAAACTCTGGGAGGCGTATACTCAAATTTCACTC |
| 6_1312-OFF(-1)-r1 | GAGTGAAATTTGAGTATACGCCTCCCAGAGTTTTATTTAAAATTTC |
| 6_1312-MASCR1 | CGTTCCATTCATCAGGGACTATC |
| 6_1312-MASCmF2 | GAAATTTTAAATAAAACTCTGGGAGCC |
| 6_1312-r1E | GGGGAATTCACCTAGGTAGACTATTTTTGC |
| 6_1312-MASCmF1 | GAAATTTTAAATAAAACTCTGGGCGCA |
| 6_1325-f1E | GGGGAATTCGGAGTAAGCATAGCTCATAAG |
| 6_1325-ON-f1 | CTTTAAAATTCAAACTTTAGGCGGAGGGTATCACAAAAAATTGGC |
| 6_1325-ON-r1 | GCCAATTTTTTGTGATACCCTCCGCCTAAAGTTTGAATTTTAAAG |
| 6_1325-OFF(-1)-f1 | CTTTAAAATTCAAACTTTAGGAGGCGGTATCACAAAAAATTGGC |
| 6_1325-OFF(-1)-r1 | GCCAATTTTTTGTGATACCCTCCGCCTAAAGTTTGAATTTTAAAG |
| 6_1325-MASCR1 | CCATACTCTCATTGATATCTGTTC |
| 6_1325-MASCmF2 | CTTTAAAATTCAAACTTTAGGAGCC |
| 6_1325-MASCmF1 | CTTTAAAATTCAAACTTTAGGCGCA |
| 6_1325-r3E | GGGGAATTCAGCTCCTTGGTATGTGGATG |
| 6_1327-f1E | GGGGAATTCGCTTTGCGATTGTCTTTGAAAAC |
| 6_1327-ON-f1 | GCAGATTTACCAAAAATTTATGGCGGAGGGTCTTATGGAGGATAC |
| 6_1327-ON-r1 | GTATCCTCCATAAGACCCTCCGCCATAAATTTTTGGTAAATCTGC |
| 6_1327-OFF(-1)-f1 | GCAGATTTACCAAAAATTTATGGAGGCGGTCTTATGGAGGATAC |
| 6_1327-OFF(-1)-r1 | GTATCCTCCATAAGACCGCCTCCATAAATTTTTGGTAAATCTGC |
| 6_1327-MASCR1 | CTATCAGTAATCCTCATGCCATG |
| 6_1327-MASCmF2 | GCAGATTTACCAAAAATTTATGGAGCC |
| 6_1327-MASCmF1 | GCAGATTTACCAAAAATTTATGGCGCA |
| 6_1327-r2E | GGGGAATTCTGGCTTTGCCAAATGAGGGTT |
| 6_1341-f1E | GGGGAATTCTGAACTTACCGCTGTTATCGC |
| 6_1341-ON-f1 | GCTCCTTTTGATACTATGGGCGGAGGTATACCAGTTATCATGATAGG |
| 6_1341-ON-r1 | CCTATCATGATAACTGGTATACCTCCGCCCATAGTATCAAAAGGAGC |
| 6_1341-OFF(-1)-f1 | GCTCCTTTTGATACTATGGGAGGCGTATACCAGTTATCATGATAGG |
| 6_1341-OFF(-1)-r1 | CCTATCATGATAACTGGTATACGCCTCCCATAGTATCAAAAGGAGC |
| 6_1341-MASCR1 | CATCAGGTGGAGCTATTTCTTTATC |
| 6_1341-MASCmF2 | GCTCCTTTTGATACTATGGGAGCC |
| 6_1341-MASCmF1 | GCTCCTTTTGATACTATGGGCGCA |
| 6_1341-r3E | GGGGAATTCTTTTAGCACCACGATTGGTTG |
| 81176_1160-f1E | GGGGAATTCTAGGTGCGGTTGCACTTGGTG |
| 6_1160-ON-f1 | GGAAATTATGGATTCATAGGCGGAGGGGATCAACTCTTG |
| 6_1160-ON-r1 | CAAGAGTTGATCCCCTCCGCCTATGAATCCATAATTTCC |
| 81176_1160-OFF(-1)-f1 | GGAAATTATGGATTCATAGGAGGCGGGATCAACTCTTG |
| 81176_1160-OFF(-1)-r1 | CAAGAGTTGATCCCGCCTCCTATGAATCCATAATTTCC |
| 81176_1160-MASCR1 | ACAGGGCGTTTTAGCATGGC |
| 81176_1160-MASCmF1 | AATAGTGGAAATTATGGATTCATAGGAGCC |
| 6_1160-MASCmF1 | AATAGTGGAAATTATGGATTCATAGGCGCA |
| 81176_1160-r1E | GGGGAATTCTTGATGCCTTTGTACATAGGG |
| 81176_1419-f1E | GGGGAATTCATTTGTCCTATATGCTGGGTG |
| 6_1419-ON-f1 | CGTATATTGACAGGCGGAGGGTATTTTACCGCGATTTGG |
| 6_1419-ON-r1 | CCAAATCGCGGTAAAATACCCTCCGCCTGTCAATATACG |
| 81176_1419-OFF(-1)-f1 | CGTATATTGACAGGAGGCGGTATTTTACCGCGATTTGG |
| 81176_1419-OFF(-1)-r1 | CCAAATCGCGGTAAAATACCGCCTCCTGTCAATATACG |
| 81176_1419-MASCR1 | TGCCACTGCTTACACGAGCA |
| 81176_1419-MASCmF1 | CCAGAATTTAATCGTATATTGACAGGAGCC |
| 6_1419-MASCmF1 | CCAGAATTTAATCGTATATTGACAGGCGCA |
| 81176_1419-r1E | GGGGAATTCTGGCTTTATCTCCTAAAACCC |
| 81176_1421-f1E | GGGGAATTCCTTTGCATACATACTCAGATG |
| 6_1421-ON-f1 | GCTATGATTGAGTTTACAAACAATGGCGGAGGGTATATAGC |
| 6_1421-ON-r1 | GCTATATACCCTCCGCCATTGTTTGTAAACTCAATCATAGC |
| 6_1421-OFF(-1)-f1 | GCTATGATTGAGTTTACAAACAATGGAGGCGGTATATAGC |
| 6_1421-OFF(-1)-r1 | GCTATATACCGCCTCCATTGTTTGTAAACTCAATCATAGC |
| 81176_1421-MASCR1 | GCCTTGATTAAAACTTCACCCAGCA |
| 6_1421-MASCmF2 | TGATTGAGTTTACAAACAATGGAGCC |
| 6_1421-MASCmF1 | TGATTGAGTTTACAAACAATGGCGCA |
| 81176_1429-f2E | GGGGAATTCAGATGATGGTGTTGCTACAAG |
| 6_1429-ON-f1 | GTAATGTATAATGGCGGAGGGTATATGAGCAATATTG |
| 6_1429-ON-r1 | CAATATTGCTCATATACCCTCCGCCATTATACATTAC |
| 6-1429-OFF(-1)-f1 | GTAATGTATAATGGAGGCGGTATATGAGCAATATTG |
| 6-1429-OFF(-1)-r1 | CAATATTGCTCATATACCGCCTCCATTATACATTAC |
| 81176_1429-MASCR1 | AACCCCATCTTGCTCTTCAGGA |
| 81176_1429-MASCmF2 | GATGGTGGTTATGTAATGTATAATGGAGCC |
| 6_1429-MASCmF1 | GATGGTGGTTATGTAATGTATAATGGCGCA |
| 81176_1429-r2E | GGGGAATTCGGAGCTCCAACTAAAGCAGCC |
| 81176_1432-f3E | GGGGAATTCTGGATGGGGTTGAAGTGGTC |
| 6_1432-ON-f1 | GCTAGTAAGAATTGGTATGGCGGAGGGTATATCAAGTTGC |
| 6_1432-ON-r1 | GCAACTTGATATACCCTCCGCCATACCAATTCTTACTAGC |
| 81176_1432-OFF(-1)-f1 | GCTAGTAAGAATTGGTATGGAGGCGGTATATCAAGTTGC |
| 81176_1432-OFF(-1)-r1 | GCAACTTGATATACCGCCTCCATACCAATTCTTACTAGC |
| 81176_1432-MASCR2 | AAACTGTAAACTTAAGTCATGTTGGTGTAC |
| 81176_1432-MASCmF1 | ATTAAAGCTAGTAAGAATTGGTATGGAGCC |
| 6_1432-MASCmF1 | ATTAAAGCTAGTAAGAATTGGTATGGCGCA |
| 81176_1432-r2E | GGGGAATTCCATTACATAACCACCATCTCC |
| 81176_1435-f1E | GGGGAATTCAAATTTGGGCGCTGTTTATGC |
| 6_1435-ON-f1 | GCTATGATTGAGTTTACAAACAATGGCGGAGGGTATATAGC |
| 6_1435-ON-r1 | GCTATATACCCTCCGCCATTGTTTGTAAACTCAATCATAGC |
| 81176_1435-OFF(-1)-f1 | GCTATGATTGAGTTTACAAACAATGGAGGCGGTATATAGC |
| 81176_1435-OFF(-1)-r1 | GCTATATACCGCCTCCATTGTTTGTAAACTCAATCATAGC |
| 81176_1435-MASCR3 | TGAAAAGCTTTCTCTCCTGTTCCATG |
| 81176_1435-MASCmF1 | CTATGATTGAGTTTACAAACAATGGAGCC |
| 6_1435-MASCmF1 | CTATGATTGAGTTTACAAACAATGGCGCA |
| 81176_1435-r1E | GGGGAATTCCTTGCTATGACAACAGCCACG |

*Single lines and double lines indicate edited bases and *Eco*RI sites for methylation, respectively.

**Table S3.** Specific combinations of template DNA and primers used to amplify the donor DNA templates.

| First-step PCR | | Second-step PCR | | Generated PCR product |
| --- | --- | --- | --- | --- |
| Template | Primers | Template | Primers |  |
| gDNA 81-176 | 6_0086-f1E and 6_0086-ON-r1 | First-step PCR products | 6_0086-f1E and 6_0086-r1E | *CJJ81176_0086*^ON^ |
| gDNA 81-176 | 6_0086-ON-f1 and 6_0086-r1E |  |  |  |
| gDNA 81-176 | 6_0086-f1E and 6_0086-OFF(-1)-r1 | First-step PCR products | 6_0086-f1E and 6_0086-r1E | *CJJ81176_0086*^OFF^ |
| gDNA 81-176 | 6_0086-OFF(-1)-f1 and 6_0086-r1E |  |  |  |
| gDNA 81-176 | 6_0206-f1E and 6_0206-ON-r1 | First-step PCR products | 6_0206-f1E and 6_0206-r1E | *CJJ81176_0206*^ON^ |
| gDNA 81-176 | 6_0206-ON-f1 and 6_0206-r1E |  |  |  |
| gDNA 81-176 | 6_0206-f1E and 6_0206-OFF(-1)-r1 | First-step PCR products | 6_0206-f1E and 6_0206-r1E | *CJJ81176_0206*^OFF^ |
| gDNA 81-176 | 6_0206- OFF(-1)-f1 and 6_0206-r1E |  |  |  |
| gDNA 81-176 | 6_0646-f1E and 6_0646-ON-r1 | First-step PCR products | 6_0646-f1E and 6_0646-r1E | *CJJ81176_0646*^ON^ |
| gDNA 81-176 | 6_0646-ON-f1 and 6_0646-r1E |  |  |  |
| gDNA 81-176 | 6_0646-f1E and 6_0646-OFF(-1)-r1 | First-step PCR products | 6_0646-f1E and 6_0646-r1E | *CJJ81176_0646*^OFF^ |
| gDNA 81-176 | 6_0646-OFF(-1)-f1 and 6_0646-r1E |  |  |  |
| gDNA 81-176 | 6_0708-f1E and 6_0708-ON-r1 | First-step PCR products | 6_0708-f1E and 6_0708-r1E | *CJJ81176_0708*^ON^ |
| gDNA 81-176 | 6_0708-f1E and 6_0708-r1E |  |  |  |
| gDNA 81-176 | 6_0708-f1E and 6_0708-OFF(-1)-r1 | First-step PCR products | 6_0708-f1E and 6_0708-r1E | *CJJ81176_0708*^OFF^ |
| gDNA 81-176 | 6_0708-OFF(-1)-r1 and 6_0708-r1E |  |  |  |
| gDNA 81-176 | 6_0758-f1E and 6_0758-ON-r1 | First-step PCR products | 6_0758-f1E and 6_0758-r1E | *CJJ81176_0758*^ON^ |
| gDNA 81-176 | 6_0758-ON-f1 and 6_0758-r1E |  |  |  |
| gDNA 81-176 | 6_0758-f1E and 6_0758-OFF(-1)-r1 | First-step PCR products | 6_0758-f1E and 6_0758-r1E | *CJJ81176_0758*^OFF^ |
| gDNA 81-176 | 6_0758- OFF(-1)-f1 and 6_0758-r1E |  |  |  |
| gDNA 81-176 | 81176_1160-f1E and 6_1160-ON-r1 | First-step PCR products | 81176_1160-f1E and 81176_1160-r1E | *CJJ81176_1160*^ON^ |
| gDNA 81-176 | cj1437c -ON-f1 and 6_1160-ON-f1 |  |  |  |
| gDNA 81-176 | 81176_1160-f1E and 81176_1160-OFF(-1)-r1 | First-step PCR products | 81176_1160-f1E and 81176_1160-r1E | *CJJ81176_1160*^OFF^ |
| gDNA 81-176 | 81176_1160-OFF(-1)-f1 and 81176_1160-r1E |  |  |  |
| gDNA 81-176 | 6_1312-f1E and 6_1312-ON-r1 | First-step PCR products | 6_1312-f1E and 6_1312-r1E | *CJJ81176_1312*^ON^ |
| gDNA 81-176 | 6_1312-ON-f1 and 6_1312-r1E |  |  |  |
| gDNA 81-176 | 6_1312-f1E and 6_1312-OFF(-1)-r1 | First-step PCR products | 6_1312-f1E and 6_1312-r1E | *CJJ81176_1312*^OFF^ |
| gDNA 81-176 | 6_1312-OFF(-1)-f1 and 6_1312-r1E |  |  |  |
| gDNA 81-176 | 6_1325-f1E and 6_1325-ON-r1 | First-step PCR products | 6_1325-f1E and 6_1325-r3E | *CJJ81176_1325*^ON^ |
| gDNA 81-176 | 6_1325-ON-f1 and 6_1325-r3E |  |  |  |
| gDNA 81-176 | 6_1325-f1E and 6_1325-OFF(-1)-r1 | First-step PCR products | 6_1325-f1E and 6_1325-r3E | *CJJ81176_1325*^OFF^ |
| gDNA 81-176 | 6_1325-OFF(-1)-f1 and 6_1325-r3E |  |  |  |
| gDNA 81-176 | 6_1327-f1E and 6_1327-ON-r1 | First-step PCR products | 6_1327-f1E and 6_1327-r2E | *CJJ81176_1327*^ON^ |
| gDNA 81-176 | 6_1327-ON-f1 and 6_1327-r2E |  |  |  |
| gDNA 81-176 | 6_1327-f1E and 6_1327-OFF(-1)-r1 | First-step PCR products | 6_1327-f1E and 6_1327-r2E | *CJJ81176_1327*^OFF^ |
| gDNA 81-176 | 6_1327-OFF(-1)-f1 and 6_1327-r2E |  |  |  |
| gDNA 81-176 | 6_1341-f1E and 6_1341-ON-r1 | First-step PCR products | 6_1341-f1E and 6_1341-r3E | *CJJ81176_1341*^ON^ |
| gDNA 81-176 | 6_1341-ON-f1 6_1341-r3E |  |  |  |
| gDNA 81-176 | 6_1341-f1E and 6_1341-OFF(-1)-r1 | First-step PCR products | 6_1341-f1E and 6_1341-r3E | *CJJ81176_1341*^OFF^ |
| gDNA 81-176 | 6_1341-OFF(-1)-f1 and 6_1341-r3E |  |  |  |
| gDNA 81-176 | 81176_1419-f1E and 6_1419-ON-r1 | First-step PCR products | 81176_1419-f1E and 81176_1419-r1E | *CJJ81176_1419*^ON^ |
| gDNA 81-176 | 6_1419-ON-f1 and 81176_1419-r1E |  |  |  |
| gDNA 81-176 | 81176_1419-f1E and 81176_1419-OFF(-1)-r1 | First-step PCR products | 81176_1419-f1E and 81176_1419-r1E | *CJJ81176_1419*^OFF^ |
| gDNA 81-176 | 81176_1419-OFF(-1)-f1 and 81176_1419-r1E |  |  |  |
| gDNA 81-176 | 81176_1421-f1E and 6_1421-ON-r1 | First-step PCR products | 81176_1421-f1E and 81176_1421-r1E | *CJJ81176_1421*^ON^ |
| gDNA 81-176 | 6_1421-ON-f1 and 81176_1421-r1E |  |  |  |
| gDNA 81-176 | 81176_1421-f1E and 6_1421-OFF(-1)-r1 | First-step PCR products | 81176_1421-f1E and 81176_1421-r1E | *CJJ81176_1421*^OFF^ |
| gDNA 81-176 | 6_1421-OFF(-1)-f1 and 81176_1421-r1E |  |  |  |
| gDNA 81-176 | 81176_1429-f2E and 6_1429-ON-r1 | First-step PCR products | 81176_1429-f2E and 81176_1429-r2E | *CJJ81176_1429*^ON^ |
| gDNA 81-176 | 6_1429-ON-f1 and 81176_1429-r2E |  |  |  |
| gDNA 81-176 | 81176_1429-f2E and 6-1429-OFF(-1)-r1 | First-step PCR products | 81176_1429-f2E and 81176_1429-r2E | *CJJ81176_1429*^OFF^ |
| gDNA 81-176 | 6-1429-OFF(-1)-f1 and 81176_1429-r2E |  |  |  |
| gDNA 81-176 | 81176_1432-f3E and 6_1432-ON-r1 | First-step PCR products | 81176_1432-f3E and 81176_1432-r2E | *CJJ81176_1432*^ON^ |
| gDNA 81-176 | 6_1432-ON-f1 and 81176_1432-r2E |  |  |  |
| gDNA 81-176 | 81176_1432-f3E and 81176_1432-OFF(-1)-r1 | First-step PCR products | 81176_1432-f3E and 81176_1432-r2E | *CJJ81176_1432*^OFF^ |
| gDNA 81-176 | 81176_1432-OFF(-1)-f1 and 81176_1432-r2E |  |  |  |
| gDNA 81-176 | 81176_1435-f1E and 6_1435-ON-r1 | First-step PCR products | 81176_1435-f1E and 81176_1435-r1E | *CJJ81176_1435*^ON^ |
| gDNA 81-176 | 6_1435-ON-f1 and 81176_1435-r1E |  |  |  |
| gDNA 81-176 | 81176_1435-f1E and 81176_1435-OFF(-1)-r1 | First-step PCR products | 81176_1435-f1E and 81176_1435-r1E | *CJJ81176_1435*^OFF^ |
| gDNA 81-176 | 81176_1435-OFF(-1)-f1 and 81176_1435-r1E |  |  |  |
| gDNA 81-176 | cjj81176_1339-f1E and flaA81176-cat-r1 | First-step PCR products | cjj81176_1339-f1E and cjj81176_1339-r1E | Δ*flaA*::*cat* |
| pSYC-*cat* | c-cat-f1 and c-cat-r2 |  |  |  |
| gDNA 81-176 | cat-flaA81176-f2 and cjj81176_1339-r1E |  |  |  |
| gDNA 81-176 | cjj81176_1339-f1E and flaA81176-kan-f1 | First-step PCR products | cjj81176_1339-f1E and cjj81176_1339-r1E | Δ*flaA*::*kan* |
| pSYC-*kan* | c-kan-f1 and c-kan-r1 |  |  |  |
| gDNA 81-176 | kan-flaA81176-f1 and cjj81176_1339-r1E |  |  |  |
| gDNA 81-176 | cjj81176_1339-f1E and cjj81176_1339-r1E |  |  | *flaA*^+^ |
| gDNA 81-176 | 176_1439-f1E and 176_1439-kan-r1 | First-step PCR products | and | Δ*kpsE*::*kan* |
| pSYC-*kan* | c-kan-f1 and c-kan-r1 |  |  |  |
| gDNA 81-176 | kan-176_1439-f1 and 176_1439-r1E |  |  |  |

**Table S4.** Specific combinations of donor DNA molecules and recipient strains used for natural transformation.

| Donor DNA (PCR fragment) | Recipient strain | Resulting strain |
| --- | --- | --- |
| Δ*flaA*::*cat* or Δ*flaA*::*kan*, *CJJ81176_0086*^OFF^, *CJJ81176_0206*^OFF^, *CJJ81176_0646*^OFF^, *CJJ81176_0708*^OFF^, *CJJ81176_0758*^OFF^, *CJJ81176_1160*^OFF^, *CJJ81176_1312*^OFF^, *CJJ81176_1325*^OFF^, *CJJ81176_1327*^OFF^, *CJJ81176_1341*^OFF^, *CJJ81176_1419*^OFF^, *CJJ81176_1421*^OFF^, *CJJ81176_1429*^OFF^, *CJJ81176_1432*^OFF^, *CJJ81176_1435*^OFF^ | 81-176 | SYC2-0K |
| *flaA*^+^ | SYC2-0K | SYC2-0 |
| Δ*flaA*::*cat*, *CJJ81176_0206*^OFF^, *CJJ81176_1325*^OFF^, *CJJ81176_1341*^OFF^ | SYC2-SV1 | SYC2-SV2C |
| *flaA*^+^ | SYC2-SV2C | SYC2-SV2 |
| *flaA*^+^, *CJJ81176_1419*^OFF^ | SYC2-SV2C | SYC2-SV3 |
| *flaA*^+^, *CJJ81176_1429*^OFF^ | SYC2-SV2C | SYC2-SV4 |
| *flaA*^+^, *CJJ81176_1435*^OFF^ | SYC2-SV2C | SYC2-SV5 |
| *flaA*^+^, *CJJ81176_0206*^OFF^, *CJJ81176_1325*^OFF^, *CJJ81176_1341*^OFF^, *CJJ81176_1419*^OFF^, *CJJ81176_1429*^OFF^, *CJJ81176_1435*^OFF^ | SYC2-SV2C | SYC2-SV6 |
| Δ*kpsE*::*kan* | 81-176 | SYC2005 |

**Table S5.** Primer mixes used for MASC PCR.

| Primer pair | Product size (bp) | ON/OFF Phase detected |  |
| --- | --- | --- | --- |
| Mix ON1 | | |  |
| 6-0758_MASC-F1 and 6-0758_MASC_ON-R1 | 540 | *CJJ81176_0758*^ON^ |  |
| 6_0708-MASCmF1 and 6_0708-MASCR1 | 417 | *CJJ81176_0708*^ON^ |  |
| 6_0206-MASCmF1 and 6_0206-MASCR1 | 300 | *CJJ81176_0206*^ON^ |  |
| 6_0646-MASCmF1 and 6_0646-MASCR1 | 210 | *CJJ81176_0646*^ON^ |  |
| 6_0086-MASCmF1 and 6_0086-MASCR1 | 104 | *CJJ81176_0086*^ON^ |  |
| Mix ON2 | | |  |
| 6_1327-MASCmF1 and 6_1327-MASCR1 | 506 | *CJJ81176_1327*^ON^ |  |
| 6_1341-MASCmF1 and 6_1341-MASCR1 | 412 | *CJJ81176_1341*^ON^ |  |
| 6_1325-MASCmF1 and 6_1325-MASCR1 | 322 | *CJJ81176_1325*^ON^ |  |
| 6_1312-MASCmF1 and 6_1312-MASCR1 | 100 | *CJJ81176_1312*^ON^ |  |
| Mix ON3 |  |  |  |
| 6_1435-MASCmF1 and 81176_1435-MASCR3 | | 709 | *CJJ81176_1435*^ON^ |
| 6_1432-MASCmF1 and 81176_1432-MASCR2 | | 512 | *CJJ81176_1432*^ON^ |
| 6_1429-MASCmF1 and 81176_1429-MASCR1 | | 406 | *CJJ81176_1429*^ON^ |
| 6_1160-MASCmF1 and 81176_1160-MASCR1 | | 332 | *CJJ81176_1160*^ON^ |
| 6_1421-MASCmF1 and 81176_1421-MASCR1 | | 221 | *CJJ81176_1421*^ON^ |
| 6_1419-MASCmF1 and 81176_1419-MASCR1 | | 145 | *CJJ81176_1419*^ON^ |
|  | | |  |
| Mix OFF1 | | |  |
| 6_0758-MASCR1 and 6_0758-MASCmF2 | 540 | *CJJ81176_0758*^OFF^ |  |
| 6_0708-MASCmF2 and 6_0708-MASCR1 | 417 | *CJJ81176_0708*^OFF^ |  |
| 6_0206-MASCmF2 and 6_0206-MASCR1 | 300 | *CJJ81176_0206*^OFF^ |  |
| 6_0646-MASCmF2 and 6_0646-MASCR1 | 210 | *CJJ81176_0646*^OFF^ |  |
| 6_0086-MASCmF2 and 6_0086-MASCR1  Mix OFF2 | 104 | *CJJ81176_0086*^OFF^ |  |
| 6_1327-MASCmF2 and 6_1327-MASCR1 | 506 | *CJJ81176_1327*^OFF^ |  |
| 6_1341-MASCmF2 and 6_1341-MASCR1 | 412 | *CJJ81176_1341*^OFF^ |  |
| 6_1325-MASCmF2 and 6_1325-MASCR1 | 322 | *CJJ81176_1325*^OFF^ |  |
| 6_1312-MASCmF2 and 6_1312-MASCR1 | 100 | *CJJ81176_1312*^OFF^ |  |
| Mix OFF3 |  |  |  |
| 81176_1435-MASCmF1 and 81176_1435-MASCR3 | 709 | *CJJ81176_1435*^OFF^ |  |
| 81176_1432-MASCmF1 and 81176_1432-MASCR2 | 512 | *CJJ81176_1432*^OFF^ |  |
| 81176_1429-MASCmF2 and 81176_1429-MASCR1 | 406 | *CJJ81176_1429*^OFF^ |  |
| 81176_1160-MASCmF1 and 81176_1160-MASCR1 | 332 | *CJJ81176_1160*^OFF^ |  |
| 6_1421-MASCmF2 and 81176_1421-MASCR1 | 221 | *CJJ81176_1421*^OFF^ |  |
| 81176_1419-MASCmF1 and 81176_1419-MASCR1 | 145 | *CJJ81176_1419*^OFF^ |  |

**Table S6.** The reference sequences used for phasevariome analysis using PVfinder_81176*.

| PVG | Locked-ON sequence | Locked-OFF sequence |
| --- | --- | --- |
| *CJJ81176_0086* | GAAGTGCATTTAACTTGGGG**C**GG**A**GTAATAGGCTTTAGGG | GAAGTGCATTTAACTTGGGG**A**GG**C**TAATAGGCTTTAGGG |
| *CJJ81176_0206* | CTAAGTATTTTAAAAATATAACCGG**C**GG**A**GGTATAGAGCCTTATGGC | CTAAGTATTTTAAAAATATAACCGG**A**GG**C**GTATAGAGCCTTATGGC |
| *CJJ81176_0646* | CCATTTAAACTAATGAGGGG**C**GG**A**GGTATTAGAACGATTTTG | CCATTTAAACTAATGAGGGG**A**GG**C**GTATTAGAACGATTTTG |
| *CJJ81176_0708* | CTTGCTATAAATTTTAATTTTACCCC**A**CC**G**CATAAAGATAAATTAG | CTTGCTATAAATTTTAATTTTACCCC**G**CC**A**ATAAAGATAAATTAG |
| *CJJ81176_0758* | CGTTTACTGACAGGCGGGG**C**GG**A**GATTTAACAATCAAACC | CGTTTACTGACAGGCGGGG**A**GG**C**ATTTAACAATCAAACC |
| *CJJ81176_1160* | GGAAATTATGGATTCATAGG**C**GG**A**GGGGATCAACTCTTG | GGAAATTATGGATTCATAGG**A**GG**C**GGGATCAACTCTTG |
| *CJJ81176_1312* | GAAATTTTAAATAAAACTCTGGG**C**GG**A**GGTATACTCAAATTTCACTC | GAAATTTTAAATAAAACTCTGGG**A**GG**C**GTATACTCAAATTTCACTC |
| *CJJ81176_1325* | CTTTAAAATTCAAACTTTAGG**C**GG**A**GGGTATCACAAAAAATTGGC | CTTTAAAATTCAAACTTTAGG**A**GG**C**GGTATCACAAAAAATTGGC |
| *CJJ81176_1327* | GCAGATTTACCAAAAATTTATGG**C**GG**A**GGGTCTTATGGAGGATAC | GCAGATTTACCAAAAATTTATGG**A**GG**C**GGTCTTATGGAGGATAC |
| *CJJ81176_1341* | GCTCCTTTTGATACTATGGG**C**GG**A**GGTATACCAGTTATCATGATAGG | GCTCCTTTTGATACTATGGG**A**GG**C**GTATACCAGTTATCATGATAGG |
| *CJJ81176_1419* | CGTATATTGACAGG**C**GG**A**GGGTATTTTACCGCGATTTGG | CGTATATTGACAGG**A**GG**C**GGTATTTTACCGCGATTTGG |
| *CJJ81176_1421* | TTTTTAAAGGAGAAACCCTATGTATAACCCAAACTCAGCTATAGAAAGAGTAAAAAATCATCTTGCTTATAAACTAGGTCAAGCTATGATTGAGTTTACAAACAATGG**C**GG**A**GGGTATATAGC | TTTTTAAAGGAGAAACCCTATGTATAACCCAAACTCAGCTATAGAAAGAGTAAAAAATCATCTTGCTTATAAACTAGGTCAAGCTATGATTGAGTTTACAAACAATGG**A**GG**C**GGTATATAGC |
| *CJJ81176_1429* | GTAATGTATAATGG**C**GG**A**GGGTATATGAGCAATATTG | GTAATGTATAATGG**A**GG**C**GGTATATGAGCAATATTG |
| *CJJ81176_1432* | GCTAGTAAGAATTGGTATGG**C**GG**A**GGGTATATCAAGTTGC | GCTAGTAAGAATTGGTATGG**A**GG**C**GGTATATCAAGTTGC |
| *CJJ81176_1435* | AAAAATAAGGAGAAACCCTATGTATAACCCAAACTCAGCTATAGAAAGAGTAAAAAATCATCTTGCTTATAAACTAGGTCAAGCTATGATTGAGTTTACAAACAATGG**C**GG**A**GGGTATATAGC | AAAAATAAGGAGAAACCCTATGTATAACCCAAACTCAGCTATAGAAAGAGTAAAAAATCATCTTGCTTATAAACTAGGTCAAGCTATGATTGAGTTTACAAACAATGG**A**GG**C**GGTATATAGC |

*Edited bases are underlined.

**Table S7.** Raw data of phasevariomes during the construction of the phase-locked library in *C. jejuni* strain 81-176.

| PVG | ON/OFF Read Counts of PVGs Across MuGENT-SSR Round | | | | | | | | | | | |  |
| --- | --- | --- | --- | --- | --- | --- | --- | --- | --- | --- | --- | --- | --- |
|  | 0 | | 1 | | 2 | | 3 | | 4 | | 5 | |  |
|  | OFF | ON | OFF | ON | OFF | ON | OFF | ON | OFF | ON | OFF | ON |  |
| *CJJ81176_0086* | 1132 | 0 | 673 | 65 | 680 | 84 | 627 | 106 | 613 | 138 | 839 | 225 |  |
| *CJJ81176_0206* | 907 | 0 | 519 | 106 | 483 | 144 | 426 | 143 | 437 | 174 | 569 | 249 |  |
| *CJJ81176_0646* | 619 | 0 | 473 | 39 | 357 | 44 | 338 | 54 | 384 | 50 | 465 | 127 |  |
| *CJJ81176_0708* | 598 | 0 | 425 | 74 | 339 | 61 | 359 | 91 | 372 | 121 | 454 | 172 |  |
| *CJJ81176_0758* | 677 | 0 | 523 | 47 | 437 | 65 | 412 | 72 | 371 | 79 | 492 | 157 |  |
| *CJJ81176_1160* | 811 | 0 | 468 | 112 | 368 | 115 | 277 | 253 | 282 | 286 | 283 | 424 |  |
| *CJJ81176_1312* | 873 | 0 | 575 | 62 | 514 | 53 | 501 | 62 | 461 | 89 | 513 | 180 |  |
| *CJJ81176_1325* | 808 | 0 | 505 | 99 | 506 | 104 | 426 | 122 | 380 | 208 | 547 | 225 |  |
| *CJJ81176_1327* | 802 | 0 | 536 | 74 | 482 | 92 | 344 | 117 | 477 | 193 | 552 | 289 |  |
| *CJJ81176_1341* | 753 | 0 | 574 | 37 | 504 | 62 | 408 | 61 | 468 | 123 | 601 | 140 |  |
| *CJJ81176_1419* | 866 | 0 | 538 | 82 | 559 | 79 | 360 | 248 | 351 | 216 | 401 | 435 |  |
| *CJJ81176_1421* | 189 | 0 | 156 | 8 | 132 | 3 | 107 | 7 | 130 | 20 | 135 | 31 |  |
| *CJJ81176_1429* | 785 | 0 | 440 | 147 | 368 | 175 | 299 | 220 | 206 | 405 | 442 | 410 |  |
| *CJJ81176_1432* | 883 | 0 | 499 | 136 | 384 | 122 | 387 | 197 | 432 | 229 | 628 | 192 |  |
| *CJJ81176_1435* | 147 | 0 | 110 | 9 | 82 | 8 | 54 | 30 | 78 | 28 | 79 | 72 |  |

**Table S8.** Raw data of phasevariomes of human serum-resistant variants from PLL_81176M.

| PVG | ON/OFF Read Counts of PVGs | | | | | | | |
| --- | --- | --- | --- | --- | --- | --- | --- | --- |
|  | Input | | Output | | | | | |
|  |  |  | 1st | | 2nd | | 3rd | |
|  | OFF | ON | OFF | ON | OFF | ON | OFF | ON |
| *CJJ81176_0086* | 840 | 213 | 771 | 129 | 793 | 98 | 814 | 81 |
| *CJJ81176_0206* | 564 | 182 | 515 | 166 | 515 | 180 | 480 | 201 |
| *CJJ81176_0646* | 402 | 123 | 341 | 133 | 440 | 64 | 388 | 35 |
| *CJJ81176_0708* | 383 | 111 | 295 | 111 | 466 | 54 | 453 | 27 |
| *CJJ81176_0758* | 409 | 121 | 236 | 339 | 74 | 483 | 60 | 479 |
| *CJJ81176_1160* | 588 | 33 | 493 | 85 | 592 | 69 | 557 | 33 |
| *CJJ81176_1312* | 569 | 129 | 564 | 122 | 608 | 60 | 642 | 60 |
| *CJJ81176_1325* | 30 | 724 | 70 | 593 | 42 | 663 | 30 | 709 |
| *CJJ81176_1327* | 615 | 116 | 473 | 181 | 685 | 59 | 611 | 50 |
| *CJJ81176_1341* | 27 | 733 | 81 | 625 | 54 | 591 | 27 | 611 |
| *CJJ81176_1419* | 820 | 49 | 139 | 621 | 29 | 714 | 26 | 729 |
| *CJJ81176_1421* | 106 | 8 | 24 | 77 | 24 | 104 | 33 | 90 |
| *CJJ81176_1429* | 78 | 547 | 401 | 184 | 525 | 176 | 464 | 189 |
| *CJJ81176_1432* | 493 | 179 | 335 | 274 | 299 | 429 | 301 | 447 |
| *CJJ81176_1435* | 104 | 13 | 36 | 69 | 9 | 95 | 2 | 113 |

**Table S9.** Raw data of phasevariomes of PLL_81176M-derived colonizing variants in the cecum and feces of IL-10-knockout mice.

| PVG | ON/OFF Read Counts of PVGs | | | | | | | | | | | | | |
| --- | --- | --- | --- | --- | --- | --- | --- | --- | --- | --- | --- | --- | --- | --- |
|  | Input | | Output | | | | | | | | | | | |
|  |  |  | m#1 | | | | m#2 | | | | m#3 | | | |
|  |  |  | Cecum | | Feces | | Cecum | | Feces | | Cecum | | Feces | |
|  | OFF | ON | OFF | ON | OFF | ON | OFF | ON | OFF | ON | OFF | ON | OFF | ON |
| *CJJ81176_0086* | 635 | 185 | 1438 | 0 | 860 | 0 | 1519 | 0 | 1430 | 1 | 722 | 0 | 712 | 0 |
| *CJJ81176_0206* | 414 | 192 | 1102 | 13 | 669 | 6 | 1259 | 9 | 1136 | 10 | 523 | 46 | 468 | 21 |
| *CJJ81176_0646* | 334 | 177 | 717 | 3 | 506 | 6 | 825 | 4 | 887 | 13 | 369 | 4 | 398 | 5 |
| *CJJ81176_0708* | 278 | 198 | 793 | 4 | 497 | 1 | 917 | 0 | 838 | 2 | 334 | 20 | 403 | 11 |
| *CJJ81176_0758* | 403 | 172 | 882 | 0 | 586 | 0 | 1043 | 0 | 919 | 0 | 424 | 0 | 415 | 2 |
| *CJJ81176_1160* | 411 | 173 | 932 | 1 | 565 | 4 | 1054 | 5 | 1032 | 9 | 492 | 4 | 493 | 12 |
| *CJJ81176_1312* | 465 | 207 | 4 | 1074 | 1 | 690 | 2 | 1240 | 1 | 1097 | 38 | 479 | 12 | 510 |
| *CJJ81176_1325* | 357 | 245 | 1083 | 0 | 633 | 0 | 1172 | 0 | 1194 | 0 | 510 | 0 | 541 | 0 |
| *CJJ81176_1327* | 390 | 261 | 1076 | 3 | 649 | 2 | 1108 | 4 | 1111 | 9 | 471 | 7 | 541 | 6 |
| *CJJ81176_1341* | 473 | 166 | 967 | 3 | 562 | 0 | 1165 | 11 | 1105 | 2 | 442 | 1 | 537 | 6 |
| *CJJ81176_1419* | 389 | 300 | 4 | 1287 | 4 | 780 | 2 | 1279 | 1 | 1166 | 42 | 554 | 13 | 568 |
| *CJJ81176_1421* | 80 | 39 | 3 | 203 | 1 | 116 | 2 | 208 | 1 | 224 | 4 | 87 | 2 | 103 |
| *CJJ81176_1429* | 191 | 431 | 5 | 1027 | 2 | 609 | 6 | 1134 | 4 | 1135 | 4 | 520 | 10 | 557 |
| *CJJ81176_1432* | 340 | 319 | 1048 | 0 | 620 | 0 | 1150 | 0 | 1128 | 1 | 481 | 0 | 565 | 4 |
| *CJJ81176_1435* | 107 | 20 | 193 | 0 | 97 | 0 | 219 | 0 | 201 | 4 | 95 | 0 | 93 | 1 |

**Table S10.** Phasotyping of single colonies isolated from pooled murine output populations.

| PVG | Phasotype* | | | | |
| --- | --- | --- | --- | --- | --- |
|  | SCm#1 | SCm#2 | SCm#3 | SCm#4 | SCm#5 |
| *CJJ81176_0086* | 0 | 0 | 0 | 0 | 0 |
| *CJJ81176_0206* | 0 | 0 | 0 | 0 | 0 |
| *CJJ81176_0646* | 0 | 0 | 0 | 0 | 0 |
| *CJJ81176_0708* | 0 | 0 | 0 | 0 | 0 |
| *CJJ81176_0758* | 0 | 0 | 0 | 0 | 0 |
| *CJJ81176_1160* | 0 | 0 | 0 | 0 | 0 |
| *CJJ81176_1312* | 1 | 1 | 1 | 1 | 1 |
| *CJJ81176_1325* | 0 | 0 | 0 | 0 | 0 |
| *CJJ81176_1327* | 0 | 0 | 0 | 0 | 0 |
| *CJJ81176_1341* | 0 | 0 | 0 | 0 | 0 |
| *CJJ81176_1419* | 1 | 1 | 1 | 1 | 1 |
| *CJJ81176_1421* | 1 | 1 | 1 | 1 | 1 |
| *CJJ81176_1429* | 1 | 1 | 1 | 1 | 1 |
| *CJJ81176_1432* | 0 | 0 | 0 | 0 | 0 |
| *CJJ81176_1435* | 0 | 0 | 0 | 0 | 0 |
|  | 284 | 284 | 284 | 284 | 284 |

* Five single colonies (SCm#1 to SCm#5) were randomly isolated from the pooled output population recovered from the ceca of three mice, and their genome sequences were analyzed to determine their phasotypes. The phase status of each gene is represented in binary format (ON = 1, OFF = 0). Phasotypes were further converted from binary to decimal.

**Table S11.** Raw data of phasevariome of PLL_81176M-derived variants recovered from chicken cecal droppings.

| PVG | ON/OFF Read Counts of PVGs | | | | | | | |
| --- | --- | --- | --- | --- | --- | --- | --- | --- |
|  | input | | Output | | | | | |
|  |  |  | c#1 | | c#2 | | c#3 | |
|  | OFF | ON | OFF | ON | OFF | ON | OFF | ON |
| *CJJ81176_0086* | 784 | 247 | 466 | 226 | 6 | 1336 | 791 | 0 |
| *CJJ81176_0206* | 535 | 260 | 593 | 0 | 8 | 1127 | 690 | 0 |
| *CJJ81176_0646* | 455 | 259 | 144 | 280 | 1 | 695 | 432 | 0 |
| *CJJ81176_0708* | 411 | 285 | 27 | 369 | 4 | 757 | 495 | 0 |
| *CJJ81176_0758* | 439 | 237 | 482 | 1 | 872 | 0 | 0 | 543 |
| *CJJ81176_1160* | 598 | 221 | 371 | 150 | 5 | 950 | 627 | 0 |
| *CJJ81176_1312* | 558 | 284 | 9 | 630 | 1012 | 5 | 674 | 0 |
| *CJJ81176_1325* | 535 | 279 | 189 | 332 | 0 | 931 | 0 | 592 |
| *CJJ81176_1327* | 542 | 376 | 372 | 151 | 934 | 7 | 0 | 654 |
| *CJJ81176_1341* | 608 | 203 | 582 | 0 | 913 | 0 | 589 | 0 |
| *CJJ81176_1419* | 508 | 287 | 394 | 200 | 3 | 1118 | 0 | 712 |
| *CJJ81176_1421* | 137 | 28 | 23 | 1 | 0 | 193 | 0 | 107 |
| *CJJ81176_1429* | 258 | 563 | 0 | 519 | 0 | 942 | 0 | 611 |
| *CJJ81176_1432* | 446 | 411 | 175 | 331 | 0 | 1021 | 669 | 0 |
| *CJJ81176_1435* | 116 | 62 | 0 | 21 | 1 | 151 | 104 | 0 |

**References**

1. J. A. Korlath, M. T. Osterholm, L. A. Judy, J. C. Forfang, R. A. Robinson, A point-source outbreak of campylobacteriosis associated with consumption of raw milk. *J. Infect. Dis.* **152**, 592–596 (1985).

2. S. Yamamoto, S. Iyoda, M. Ohnishi, Stabilizing Genetically Unstable Simple Sequence Repeats in the *Campylobacter jejuni* Genome by Multiplex Genome Editing: a Reliable Approach for Delineating Multiple Phase-Variable Genes. *mBio.* **12**, e0140121 (2021).
